# Design, Synthesis and Biological Evaluation of Novel Primaquine-Cinnamic Acid Conjugates of the Amide and Acylsemicarbazide Type

**DOI:** 10.3390/molecules21121629

**Published:** 2016-11-28

**Authors:** Kristina Pavić, Ivana Perković, Petra Gilja, Filip Kozlina, Katja Ester, Marijeta Kralj, Dominique Schols, Dimitra Hadjipavlou-Litina, Eleni Pontiki, Branka Zorc

**Affiliations:** 1 Faculty of Pharmacy and Biochemistry, University of Zagreb, A. Kovačića 1, HR-10 000 Zagreb, Croatia; kpavic@pharma.hr (K.P.); iperkovic@pharma.hr (I.P.); pgilja@student.pharma.hr (P.G.); filip.kozlina@gmail.com (F.K.); 2 Division of Molecular Medicine, Rudjer Bošković Institute, Bijenička cesta 54, HR-10 000 Zagreb, Croatia; katja.ester@irb.hr (K.E.); marijeta.kralj@irb.hr (M.K.); 3 Rega Institute for Medical Research, Katholieke Universiteit Leuven, Minderbroedersstraat 10, B-3000 Leuven, Belgium; dominique.schols@rega.kuleuven.be; 4 Faculty of Health Sciences, School of Pharmacy, Aristotles University of Thessaloniki, Thessaloniki 54 124, Greece; hadjipav@pharm.auth.gr (D.H.-L.); epontiki@pharm.auth.gr (E.P.)

**Keywords:** primaquine, cinnamic acid derivative, conjugate, cytostatic activity, antiviral activity, antioxidative activity

## Abstract

In this paper design and synthesis of a scaffold comprising primaquine (PQ) motif and cinnamic acid derivatives (CADs) bound directly (compounds **3a**–**k**) or via a spacer (compounds **7a**–**k**) are reported. In the first series of compounds, PQ and various CADs were connected by amide bonds and in the second series by acylsemicarbazide functional groups built from the PQ amino group, CONHNH spacer and the carbonyl group originating from the CADs. PQ-CAD amides **3a**–**k** were prepared by a simple one-step condensation reaction of PQ with a series of CAD chlorides (method A) or benzotriazolides **2** (method B). The synthesis of acylsemicarbazides **7a**–**k** included activation of PQ with benzotriazole, preparation of PQ-semicarbazide **6** and its condensation with CAD chlorides **4**. All synthesized PQ-CAD conjugates were evaluated for their anticancer, antiviral and antioxidative activities. Almost all compounds from series **3** were selective towards the MCF-7 cell line and active at micromolar concentrations. The *o*-fluoro derivative **3h** showed high activity against HeLa, MCF-7 and in particular against the SW 620 cell line, while acylsemicarbazide **7f** with a benzodioxole ring and **7c**, **7g** and especially **7j** with methoxy-, chloro- or trifluoromethyl-substituents in the *para* position showed high selectivity and high inhibitory activity against MCF-7 cell line at micromolar (**7c**, **7f**, **7g**) and nanomolar (**7j**) levels. Acylsemicarbazide derivatives with trifluoromethyl group(s) **7i**, **7j** and 7**k** showed specific activity against human coronavirus (229E) at concentrations which did not alter the normal cell morphology. The same compounds exerted the most potent reducing activity in the DPPH test, together with **7d** and **7g**, while methoxy (compounds **7c**–**e**), benzodioxole (**7f**), *p*-Cl (**7g**) and *m*-CF_3_ (**7i**) acylsemicarbazides and amide **3f** presented the highest LP inhibition (83%–89%). The dimethoxy derivative **7d** was the most potent LOX inhibitor (IC_50_ = 10 μΜ). The performed biological tests gave evidence of acylsemicarbazide functional group as superior binding group in PQ-CAD conjugates.

## 1. Introduction

The molecular hybridization approach based on the combination of the pharmacophoric moieties of different compounds was used to produce new hybrid molecules with cinnamic acid derivatives (CADs) and primaquine (PQ) motifs. Cinnamic acid (CA, *trans*-3-phenylacrylic acid, *trans*-3-phenyl-2-propenoic acid) and its derivatives are naturally occurring substances found in various plants. They are important intermediates in biosynthetic pathways of secondary metabolites, which play key roles in plant growth, development, reproduction and disease resistance [1]. Numerous research papers report various pharmacological activities of CADs [2,3]. First of all, they exhibit strong antimicrobial, antifungal and antiviral activity [4,5,6,7,8]. A review on natural and synthetic CADs with antimicrobial activity has been recently published [1]. CADs show good to moderate antitubercular activity or synergistic activity with antitubercular drugs [9,10,11,12,13]. Many CADs, especially those with phenolic hydroxyl groups, are well-known antioxidants and are supposed to have several health benefits due to their strong free radical scavenging properties [14,15,16]. Methoxy substituted cinnamates play an important role in controlling inflammatory diseases [17], while methoxy substituted octylcinnamates are efficient sunscreen agents [18]. 3-Hydroxy, 4-hydroxy and 3,4-dihydroxy CADs possess hepatoprotective [19] and hypolipidemic activity [20,21], while halogenated derivatives have CNS depressant activity [22]. Some amides derived from CA are potential antimalarial leads [23]. *N*-cinnamoyl chloroquine, PQ, aminoacridines and 4-aminoquinoline derivatives are reported as dual-action antimalarials and antiproliferative agents [24,25,26,27]. Anticancer potential of piplartine, a CA alkaloid/imide derivative from peppers, and various CADs have been reported in numerous papers and reviewed by Bezerra [28] and De and co-workers [29]. Finally, CA motifs are present in several approved antiallergic drugs, such as cinnarizine, cinaserin and tranilast and antiplatelet agent ozagrel [30].

On the other hand, PQ is a well-known antimalarial drug with pronounced antiproliferative activity. Numerous reports corroborate anticancer properties of antimalarial drug classes and their usefulness in adjuvant chemotherapies [31,32,33,34,35,36,37,38,39,40,41]. Several antimalarials reached a clinical stage in anticancer research [42].

In several papers, we have shown that PQ derivatives of urea and acylsemicarbazide type possess strong antiproliferative effects against a number of tumor cell lines and/or high selectivity towards the breast cell line MCF-7 [43,44,45,46,47]. The most active compounds have one or more aromatic rings attached to a PQ scaffold via nitrogen- and oxygen-rich spacers (urea, carbamate, hydroxyurea, double urea). The literature survey showed that hydroxysemicarbazides are potential antitumor agents as well [48]. In the light of the above considerations, we have designed a series of hybrid molecules with cinnamoyl residue as the aromatic component bound by amide or acylsemicarbazide functionality to PQ pharmacophore. Our new series of PQ derivatives differ from the previous ones both in the aromatic region and in the type of spacers. The present study reports the synthesis and characterization of PQ-CAD conjugates and their evaluation as potential anticancer and antiviral agents. The chemopreventive potential on carcinogenesis, inhibition of lipoxygenase as a marker of anti-inflammatory activity and antioxidative ability of PQ-CAD were evaluated as well.

## 2. Results and Discussion

### 2.1. Chemistry

**Scheme 1:**
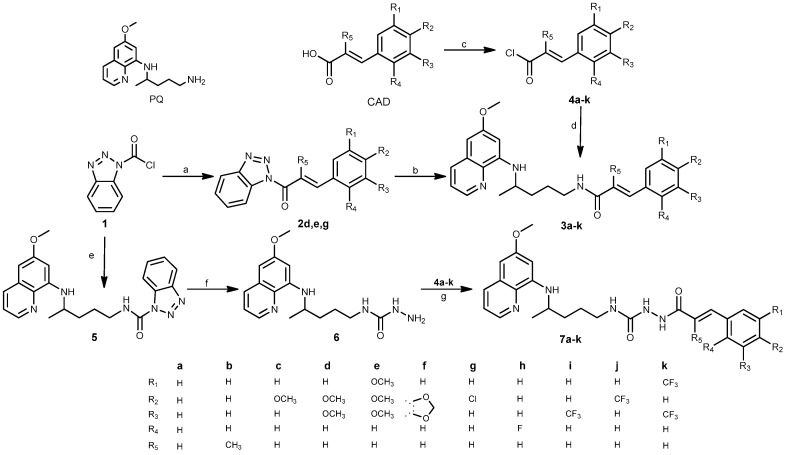
Synthetic route for preparation of compounds **3** and **7** and their precursors. *Reagents and conditions:* (**a**) CA or CAD, TEA, toluene, 0.5 h; (**b**) PQ, TEA, dioxane, 20 h; (**c**) SOCl_2_, toluene, DMF, 3 h; (**d**) PQ, TEA, CH_2_Cl_2_, 0.5–3 h; (**e**) PQ, TEA, toluene, 24 h; (**f**) NH_2_NH_2_ × H_2_O, Na_2_S_2_O_4_, dioxane, 4 days; (**g**) **4a**–**k**, TEA, CH_2_Cl_2_, overnight. All reactions were performed at r.t. The reactions with PQ were run light protected.

Two series of PQ-CAD conjugates **3a**–**k** and **7a**–**k** were prepared, with CA, α-methylcinnamic acid or methoxy, dimethoxy, trimethoxy, methylenedioxy, chloro, fluoro, trifluoromethyl and bis-trifluoromethylcinnamic acid as the CAD component. In series **3** PQ and CA Q moieties are directly bound by an amide bond, while in series **7** they are connected by the CONHNH spacer. Terminal amino group of PQ, the hydrazide spacer and CA carbonyl group together form a new acylsemicarbazide functional group. The synthetic route leading to PQ-CAD conjugates is outlined in Scheme 1.

Two methods for the preparation of amides **3a**–**k** were applied: acid chlorides **4** (method A) and benzotriazolides **2** (method B) were used as the activated CAD intermediates. Both methods gave products in similar yields. CAD-benzotriazolides **2** were prepared following our procedure described previously: benzotriazolides of various carboxylic acids were synthesized and transformed to esters, amides or hydroxamic acids [49,50,51,52]. Benzotriazolides **2** (active amides) and PQ-benzotriazolide **5** (active urea) were prepared from the same starting compound BtcCl (**1**) and CAD or PQ, in the presence of triethylamine (TEA). Reaction of **5** with hydrazine gave semicarbazide **6**, which reacted with chlorides **4** and afforded PQ-CAD conjugates of general formula **7**. Compounds **5** and **6** are stable intermediates applied in our previous work for syntheses of numerous PQ derivatives [43,46].

All new compounds were fully characterized by the usual spectroscopic methods (IR, ^1^H-, ^13^C-NMR and MS) and elemental analyses. Spectral data are consistent with the proposed structures and are summarized in the Experimental section and in detail in the Supplementary Materials. Syntheses of compounds **3a**, **3c** and **3g** were reported previously, but without any spectral and analytical data [26]. Compounds **2** and **4** were used in further reaction steps without purification and their structures were confirmed indirectly.

The presence of the PQ residue in compounds **3**, **5**, **6** and **7** was confirmed by NMR spectra: hydrogen atoms next to pyridine nitrogen occurred in ^1^H-NMR spectra at δ between 8.55 and 8.51, methoxy group at 3.81–3.80, CH of chiral carbon as a multiplet at 3.70–3.63, methyl group between 1.24 and 1.20 ppm, while in ^13^C spectra the corresponding carbon atoms appeared at 144.27–144.18, 55.18–54.91, 47.01–46.98 and 20.25–20.19 ppm, respectively. CAD benzene ring afforded additional hydrogen or carbon signals in aromatic region. Signals of methoxy groups in products **3c**–**e** and **7c**–**e** were located very close to the PQ methoxy group. CF_3_ groups present in **3i**–**k** and **7i**–**k** and adjacent aromatic carbon atoms split in ^13^C-NMR spectra and appeared as quartets at 132.28–118.69 and 131.14–128.44 ppm, respectively.

Amide NH signals in series **3** were visible in the ^1^H-NMR spectra at 8.24–7.95 and amine NH at 6.16–6.11 ppm (all D_2_O exchangeable). Compounds from series **7** showed four NH signals: the NH close to the CAD carbonyl appeared as a singlet between 9.85 and 9.62 ppm, the next NH as a singlet between 7.99 and 7.67, the PQ terminal amino group between 6.53 and 6.43 and PQ NH group next to chiral atom as doublet between 6.14 and 6.09 ppm. The presence of one (compounds **3a**–**k**) and two carbonyl groups (compounds **7a**–**k**) was indicated by the appearance of strong stretching vibration bands in the corresponding IR spectra between 1663 and 1651 cm^−1^ and signals between 168.71 and 164.07 ppm in the ^13^C-NMR spectra.

All PQ-CAD amides and almost all acylsemicarbazides are fully in agreement with the Lipinski rule of five and Gelovani rules for prospective small molecular drugs: MW ≤ 500, log *P* ≤ 5, number of H-bond donors ≤ 5, number of H-bond acceptors ≤ 10, molecular polar surface area (PSA) < 140 Å^2^ molar refractivity (MR) within the range of 40 and 130 cm^3^/mol, the number of atoms 20–70 [53]. The parameters were calculated with the Chemicalize.org program [54] and are presented in Table 1.

### 2.2. Biological Evaluation

Considering the individual biological and medicinal importance of PQ and CADs, we wanted to explore novel chemical entities based on PQ and CAD moieties with respect to their biological significance. The synthesized PQ-CAD conjugates were evaluated for their anticancer, antiviral, anti-inflammatory and antioxidative activities. The results are summarized in Table 2 and Table 3.

#### 2.2.1. Anticancer Activity

The newly prepared PQ-CAD derivatives were designed and prepared primarily as potential anticancer agents. Their antiproliferative activity was evaluated in vitro on five different types of human tumor cell lines: lymphoblastic leukemia (CEM), cervical carcinoma (HeLa), lung carcinoma (NCI-H460), colon carcinoma (SW 620), breast carcinoma (MCF-7) and murine lymphocytic leukemia (L1210) and compared with the standard anticancer drugs (sorafenib, cisplatin and 5-fluorouracil) and PQ. Amide derivatives **3a**–**k** showed weak activity against L1210 and CEM, and no activity on H460 cell line. Most of the compounds of series **3** were inactive against SW 620. However, **3a** and **3i** showed mild and **3h** very strong activity (IC_50_ 0.3 ± 0.1 μM) against the same cell line. Activity against HeLa varied very much: derivatives **3a** and **3h** showed strong activity in low micromolar concentration (IC_50_ 4.0 ± 0.9 and 2.1 ± 2.1 μM, respectively), while the other amide derivatives were either inactive (**3c**–**e**) or their activity was very weak (the rest of the compounds in series **3**) (Table 2). MCF-7 was the most susceptible tumor cell line. Practically all compounds **3** were active in low micromolar concentrations. Again, the most active was the *o*-fluoro derivative **3h** (IC_50_ 1.1 ± 0.6 μM). Acylsemicarbazide derivatives **7a**–**k** were more active than amide derivatives and more or less active against all the tested cell lines and, in general, very active against MCF-7. Four compounds, **7c**, **7g**, **7f** and **7j**, showed very strong activity against MCF-7 and variable activity against the other tested cell lines. It is interesting to note that three of the most active PQ-CAD derivatives bear substituent in *para* position (methoxy, chloro and trifluoromethyl, respectively). The most active compound was trifluoromethyl derivative **7j** with IC_50_ in nanomolar scale (30 ± 20 nM). This derivative exerted 100-to 500-fold higher activity than the anticancer reference drugs and 900-fold higher activity than PQ.

The most interesting finding of the current research is the high sensitivity of MCF-7 tumor cells to this series of compounds. We have already noticed and discussed this phenomenon in our previous work with PQ derivatives [43,45,46]. The investigation of the underlying mechanism is in progress.

#### 2.2.2. Antiviral Activity Assays

Compounds **3a**–**k** and **7a**–**k** were evaluated against a broad variety of viruses including herpes simplex virus type 1 (KOS), herpes simplex virus 2 (G), herpes simplex virus 1 TK^–^(KOS) ACV^r^, vaccinia virus, adeno virus 2 and human coronavirus (229E) in HEL cell cultures and their activities were compared with reference compounds such as brivudin, cidofovir, acyclovir, gancyclovir, zalcitabine, alovudine, *Urtica dioica* agglutinin (UDA) and ribavirin, respectively. Compounds of series **3** were inactive towards all tested viruses, while acylsemicarbazide derivatives **7b**, **7f** (EC_50_ = 15.0 and 12.5 μΜ, respectively), **7i**, **7j** and **7k** (EC_50_ = 7.9–9.5 μΜ) showed moderate activity against human coronavirus (229E), but three or two fold lower activity than UDA (EC_50_ = 4.0 μΜ). These compounds did not alter normal cell morphology in confluent HEL cell cultures at 100 µM concentration (data not shown): selectivity ratio (SI) (MCC to EC_50_ ratio, e.g., ratio of minimum cytotoxic concentration (concentration that causes a microscopically detectable alteration of normal cell morphology) and concentration required to reduce virus-induced cytopathogenicity by 50%) was close to ten. It is worth mentioning that the three most active compounds contain trifluoromethyl group: **7i** (*m*-CF_3_, EC_50_ = 7.9 μΜ), **7j** (*p*-CF_3_, EC_50_ = 9.5 μΜ), **7k** (two *m*-CF_3_, EC_50_ = 8.4 μΜ), while halogen and methoxy derivatives were inactive. Therefore, one can conclude that acylsemicarbazide functional group and the presence of CF_3_ motif may be responsible for enhancing antiviral activity against human coronavirus. These findings could be useful in further structural optimization.

#### 2.2.3. Cellular Cytotoxicity Assays

Cytotoxicity measurements were based on the inhibition of HEL growth and the results were expressed as MCC. MCC values for all PQ-CAD derivatives were equal or higher than 100 μΜ, except for **7c** and **7g**, which was around 20 μΜ.

#### 2.2.4. Antioxidative Activity

Antioxidant potentials of new PQ and CAD hybrids **3a**–**k** and 7**a**–**k** were studied and compared to the well-known antioxidant agents such as nordihydroguaiaretic acid (NDGA) and 6-hydroxy-2,5,7,8-tetramethylchroman-2-carboxylic acid (Trolox). Two different antioxidant assays were used: (i) interaction with the stable 1,1-diphenyl-2-picrylhydrazyl (DPPH) free radical; and (ii) interaction with the water-soluble azo compound 2,2’-azobis(2-amidinopropane) dihydrochloride (AAPH), used as a source of peroxyl radicals [47].

DPPH interaction of the tested compounds was examined at 50 and 100 μΜ concentrations after 20 and 60 min. DPPH-reducing ability (RA) of the amide derivatives **3a**–**k** was very low or missing at 50 µM. However, an increase was observed with the increase of their concentration to 100 μM. The highest activity was presented by the unsubstituted derivative **3a** (53%) (Table 3). On the contrary, the acylsemicarbazide derivatives **7a**–**k** exhibited stronger antioxidant activity in both concentrations and this ability was found to be concentration and time dependent for the majority of compounds. Trifluoromethyl derivatives **7j**, **7k, 7i**, and dimethoxy **7d** and chloro derivative **7g** showed the highest reducing activity within the dataset. Perusal of RA values (**3a** < **7a**, **3b** < **7b**, etc.) supported the fact that keeping the rest structural characteristics the same, the presence of acylsemicarbazide functional group was correlated with higher antioxidant activity.

In the acylsemicarbazide series **7**, the attachment of CF_3_ group(s) on the cinnamic ring enhanced the antioxidant ability. The presence of two vicinal methoxy groups *(m*, *p*) lead to higher activity, compared to the presence of single methoxy group (**7d** > **7c**). On the contrary, the third methoxy group diminished the activity (**7e**), probably due to steric reasons (bulky radical DPPH could not approach and interact). Replacement of the phenyl ring by a benzo[*d*][1,3]dioxole increased activity (**7f** > **7a**). The nature and/or position of the halogen atom on the cinnamic ring affected the antioxidant potency. Thus, *p*-Cl derivative **7g** was found more potent than *o*-F **7h**. For both groups of compounds no correlation between overall lipophilicity and activity was found (Table 1 and Table 3). However, hydrophilic contribution of the spacer seems to play a more significant role (log *P* **3a** < **7a**, **3b** < **7b**, etc.)

In our studies, AAPH was used as a free radical initiator to follow oxidative changes of linoleic acid to conjugated diene hydroperoxide. The results showed that all amide derivatives **3** significantly inhibited lipid peroxidation (LP) (32%–87%). Compound **3f** with a benzodioxole ring exhibited the highest activity. Again, acylsemicarbazides **7** generally presented higher LP inhibition (50%–89%) than amides. Unsubstituted (**7a**), methoxy (**7c**–**e**), benzodioxole (**7f**), *p*-Cl (**7g**) and *m*-CF_3_ (**7i**) derivatives were the most potent (83%–89%). A correlation between lipophilicity and LP inhibition was not found.

LOX inhibitors have attracted attention initially as potential agents for the treatment of inflammatory and allergic diseases, certain types of cancer, and cardiovascular diseases. In order to diminish the pro-cancerous mechanisms, a key treatment strategy is to reduce the free radical load. Antioxidant activity by scavenging of reactive oxygen species is important in preventing potential damage of cellular compounds such as DNA, proteins and lipids. Moreover, it has been found that LOX inhibitors may have chemopreventive activity in lung carcinogenesis [55]. Reports published over the past three decades support a growth-regulatory role of arachidonic acid metabolites in the etiology of mammary carcinogenesis [56]. Studies have demonstrated that levels of several eicosanoids are increased in breast cancer in comparison to benign breast tumours [56]. In this context, we evaluated the newly prepared PQ-CAD conjugates for their ability to inhibit soybean lipoxygenase (LOX). From the tested compounds, all amides **3**, with the exception of **3j**, were inactive (10%–45% at 100 μΜ). However, acylsemicarbazides **7** exhibited significant activity in LOX inhibition assay (IC_50_ values 10–100 μΜ). Dimethoxy derivative **7d** was the most potent inhibitor with an IC_50_ value of 10 μΜ (double as the concentration of the reference compound NDGA), followed by **7j** and **7h**. The compounds with high log *P* values were not active, so the idea that lipophilicity was an important physicochemical property for LOX inhibition was not supported. Our results indicated that LOX inhibition is accompanied and correlated with antilipid peroxidation and DPPH radical scavenging activity. In general, the presence of acylsemicarbazide moiety in PQ-CAD conjugates led to hybrids with better biological response.

## 3. Experimental Section

### 3.1. Chemistry

#### 3.1.1. Materials and Methods

Melting points were measured on Stuart Melting Point (SMP3) apparatus (Barloworld Scientific, Staffordshire, UK) in open capillaries with uncorrected values. IR spectra were recorded on FTIR Perkin Elmer Paragon 500 and UV-Vis spectra on Lambda 20 double beam spectrophotometer (Perkin-Elmer, Waltham, MA, USA). All NMR (^1^H and ^13^C) spectra were recorded at 25 °C on NMR Avance 600 (Bruker, Rheinstetten, Germany) and Varian Inova 400 spectrometers (Varian, Palo-Alto, CA, USA) at 300, 400 and 600 MHz for ^1^H and 75, 100 and 150 MHz for ^13^C nuclei, respectively. Chemical shifts (δ) are reported in parts per million (ppm) using tetramethylsilane as reference in the ^1^H and the DMSO residual peak as reference in the ^13^C spectra (39.51 ppm). Coupling constants (*J*) are reported in Hertz (Hz). Mass spectra were recorded on HPLC-MS/MS (HPLC, Agilent Technologies 1200 Series; MS, Agilent Technologies 6410 Triple Quad, Santa Clara, CA, USA). Mass determination was realized using electron spray ionization (ESI) in positive mode. Elemental analyses were performed on a CHNS LECO analyzer (LECO Corporation, St. Joseph, MI, USA). All compounds were routinely checked by TLC with Merck silica gel 60F-254 glass plates using the following solvent systems: petrolether/ethyl acetate/methanol 30:10:5, dichloromethane/methanol 97:3 and 95:5. Spots were visualized by short-wave UV light and iodine vapor. Column chromatography was performed on silica gel 0.063–0.200 mm (Kemika, Zagreb, Croatia) and 0.040–0.063 mm (Merck, Darmstadt, Germany), with the same eluents used in TLC. All chemicals, solvents and biochemical reagents were of analytical grade and purchased from commercial sources. CA, α-methylcinnamic acid ((2-methyl-3-phenyl)acrylic acid), 4-methoxy-cinnamic acid (*p*-coumaric acid methyl ether), 3,4-dimethoxycinnamic acid (caffeic acid dimethyl ether), 3,4,5-trimethoxycinnamic acid, 3,4-(methylenedioxy)cinnamic acid (3-benzo[1,3]-dioxol-5-yl-acrylic acid), 4-chlorocinnamic acid, 2-fluorocinnamic acid, 4-(trifluoromethyl)cinnamic acid, 3-(trifluoromethyl)cinnamic acid and 3,5-bis(trifluoromethyl)cinnamic acid were purchased from Sigma-Aldrich (St. Louis, MO, USA), predominantly as *trans* stereoisomers (≥99%). 1*H*-benzo[*d*][1,2,3] triazole (BtH), triphosgene, PQ diphosphate, TEA, hydrazine hydrate, LOX, linoleic acid sodium salt, DPPH, NDGA, AAPH and Trolox were purchased from Sigma-Aldrich as well. PQ was prepared from PQ diphosphate prior the use. All reactions with PQ were run light protected.

#### 3.1.2. General Procedure for the Synthesis of Benzotriazolides **2d**,**e**,**g**

To a solution of CA or CAD (1 equiv.) and TEA (1 equiv.) in dry toluene, solution of BtcCl (**1**) (1 equiv.) in dry toluene was added dropwise. The reaction mixture was stirred at room temperature for 30 min. Solvent was evaporated and the residue was dissolved in EtOAc/H_2_O mixture (1:1). The organic layer was extracted with water, dried over anhydrous sodium sulfate, filtered and evaporated.

*(E)-1-(1H-Benzo[d][1,2,3]triazol-1-yl)-3-(3,4-dimethoxyphenyl)prop-2-en-1-one* (**2d**): Compound **2d** was synthesized according to the general procedure using 3,4-dimethoxycinnamic acid (0.208 g, 1 mmol) and BtcCl (0.181 g, 1 mmol). The crude product (0.207 g, 67%) was triturated with ether and used in further reactions without purification.

*(E)-1-(1H-Benzo[d][1,2,3]triazol-1-yl)-3-(3,4,5-trimethoxyphenyl)prop-2-en-1-one* (**2e**): Compound **2e** was synthesized according to the general procedure using 3,4,5-trimethoxycinnamic acid (0.155 g, 0.65 mmol) and BtcCl (0.118 g, 0.65 mmol). The crude product (0.128 g, 58%) was triturated with ether/petrolether and used in further reactions without purification.

*(E)-1-(1H-Benzo[d][1,2,3]triazol-1-yl)-3-(4-chlorophenyl)prop-2-en-1-one* (**2g**): Compound **2g** was synthesized according to the general procedure using 4-chlorocinnamic acid (0.365 g, 2 mmol) and BtcCl (0.362 g, 2 mmol). The crude product (0.238 g, 42%) was triturated with ether and used in further reactions without purification.

#### 3.1.3. General Procedure for the Synthesis of Chlorides **4a**–**k**

A solution of CA or CAD (1 equiv.), thionyl chloride (5 equiv.) and two drops of DMF in dry toluene was stirred at room temperature for 3 h. Solvent was evaporated. The residue was dissolved in dry toluene, evaporated again and used in further reactions without purification.

#### 3.1.4. General Procedure for the Synthesis of Amides **3a**–**k**

*Method A:* A suspension of PQ diphosphate (1 equiv.) and TEA (3 equiv.) in dry dichloromethane was stirred at room temperature. After 15 min, a solution of chloride **4** (1.25 equiv.) in dry dichloromethane was added dropwise. The reaction mixture was stirred at room temperature, light protected. After 0.5–3 h, solvent was removed under reduced pressure and the residue was dissolved in ethyl acetate/5% NaOH mixture (1:1). The organic layer was extracted with 5% NaOH solution and water, dried over anhydrous sodium sulfate, filtered and evaporated. The crude product was purified by column chromatography.

*Method B:* NaOH solution was added to a solution of PQ diphosphate (1 equiv.) in water until pH 9-10 was reached and PQ base was extracted with dichloromethane. The organic layer was dried over anhydrous sodium sulfate, filtered and evaporated. PQ and TEA (1 equiv.) were dissolved in dry dioxane and added dropwise to a solution of **2** (1 equiv.) in dry dioxane. The reaction mixture was stirred at room temperature overnight and evaporated under reduced pressure. The residue was dissolved in ethyl acetate and extracted with 5% NaOH solution and water, dried over anhydrous sodium sulfate, filtered and evaporated. The crude product was purified by column chromatography.

*(E)-N-(4-(6-Methoxyquinolin-8-ylamino)pentyl)-3-phenylacrylamide* (**3a**): Compound **3a** was synthesized according to the general procedure (method A) using chloride **4a** (0.108 g, 0.65 mmol) and PQ diphosphate (0.228 g, 0.5 mmol). After purification by column chromatography (CH_2_Cl_2_/MeOH = 95:5) and recrystallization from EtOAc/ether, pure compound **3a** was obtained (0.126 g, 65%, m.p. = 72–74 °C). IR (KBr) (cm^−1^): 3387, 3282, 3061, 2927, 2852, 1660, 1614, 1576, 1556, 1516, 1454, 1424, 1387, 1338, 1220, 1203, 1160, 1052, 1031, 977, 902, 863, 822, 791, 766, 680, 626. ^1^H-NMR (δ*/*ppm): 8.55–8.53 (dd, 1H, *J* = 1.43, 4.13), 8.13 (t, 1H, *J* = 5.69), 8.09–8.06 (dd, 1H, *J* = 1.43, 8.28), 7.55 (d, 2H, *J* = 6.49), 7.45–7.34 (m, 5H), 6.61 (d, 1H, *J* = 15.82), 6.47 (s, 1H), 6.28 (s, 1H), 6.15 (d, 1H, *J* = 8.73), 3.81 (s, 3H), 3.66 (m, 1H), 3.21 (m, 2H), 1.64 (m, 4H), 1.22 (d, 3H, *J* = 6.25). ^13^C-NMR (δ*/*ppm): 164.78, 159.01, 144.64, 144.23, 138.40, 134.96, 134.80, 134.52, 129.58, 129.33, 128.89, 127.44, 122.32, 122.10, 96.12, 91.61, 54.96, 47.01, 39.02, 33.54, 26.03, 20.23. MS (ESI): *m*/*z* = 390.3 [M + H]^+^. Anal. Calcd. For C_24_H_27_N_3_O_2_: C, 74.31; H, 6.99; N, 10.79. Found: C, 74.49; H, 7.02; N, 10.50.

*(E)-N-(4-(6-Methoxyquinolin-8-ylamino)pentyl)-2-methyl-3-phenylacrylamide* (**3b**): Compound **3b** was synthesized according to the general procedure (method A) using chloride **4b** (0.117g, 0.65 mmol) and PQ diphosphate (0.228 g, 0.5 mmol). After purification by column chromatography (CH_2_Cl_2_/MeOH = 95:5), oil compound **3b** was obtained (0.157 g, 78%). IR (film) (cm^−1^): 3378, 3054, 2936, 2867, 1735, 1651, 1616, 1578, 1519, 1456, 1424, 1388, 1271, 1220, 1202, 1158, 1052, 1031, 926, 900, 822, 792, 763, 738, 703, 624. ^1^H-NMR (δ*/*ppm): 8.55–8.53 (dd, 1H, *J* = 1.63, 4.19), 8.09–8.03 (m, 2H), 7.45–7.28 (m, 6H), 7.18 (s, 1H), 6.48 (s, 1H), 6.28 (s, 1H), 6.16–6.14 (d, 1H, *J* = 8.75), 3.81 (s, 3H), 3.70–3.63 (m, 1H), 3.23–3.17 (m, 2H), 1.99 (s, 3H), 1.73–1.56 (m, 4H), 1.24–1.23 (d, 3H, *J* = 6.29). ^13^C-NMR (δ*/*ppm): 168.71, 159.02, 144.58, 144.27, 136.14, 134.80, 134.54, 132.68, 131.87, 129.58, 129.17, 128.35, 127.56, 122.10, 96.11, 91.58, 54.96, 47.04, 39.02, 33.43, 25.95, 20.19, 14.29. MS (ESI): *m*/*z* = 404.3 [M + H]^+^. Anal. Calcd. For C_25_H_29_N_3_O_2_: C, 74.41; H, 7.24; N, 10.41. Found: C, 74.20; H, 7.02; N, 10.66.

*(E)-3-(4-Methoxyphenyl)-N-(4-(6-methoxyquinolin-8-ylamino)pentyl)acrylamide* (**3c**): Compound **3c** was synthesized according to the general procedure (method A) using chloride **4c** (0.128 g, 0.65 mmol) and PQ diphosphate (0.228 g, 0.5 mmol). After purification by column chromatography (cyclohexane/EtOAc/MeOH = 30:10:7 and CH_2_Cl_2_:/MeOH = 95:5) and recrystallization from EtOAc/ether, pure compound **3c** was obtained (0.133 g, 66%, m.p. = 78–80 °C). IR (KBr) (cm^−1^): 3255, 3070, 2962, 2930, 2856, 1738, 1652, 1610, 1571, 1517, 1455, 1424, 1385, 1304, 1229, 1168, 1032, 981, 823, 791, 678, 630. ^1^H-NMR (δ*/*ppm): 8.54–8.53 (dd, 1H, *J* = 1.5, 4.2), 8.09–8.05 (dd, 1H, *J* = 1.4, 8.3), 8.02–7.98 (t, 1H, *J* = 5.4), 7.50–7.47 (d, 2H, *J* = 8.69), 7.44–7.40 (m, 1H), 7.38–7.32 (d, 1H, *J* = 15.75), 6.98–6.95 (d, 2H, *J* = 8.70), 6.48–6.42 (m, 2H), 6.28 (s, 1H), 6.15–6.12 (d, 1H, *J* = 8.74), 3.81 (s, 3H), 3.78 (s, 3H), 3.65 (m, 1H), 3.21–3.19 (m, 2H), 1.7–1.56 (m, 4H), 1.23–1.21 (d, 3H, *J* = 6.27). ^13^C-NMR (δ*/*ppm): 165.05, 160.18, 158.98, 144.61, 144.18, 138.02, 134.74, 134.49, 129.53, 128.93, 127.50, 122.03, 119.85, 114.30, 96.08, 91.61, 55.18, 54.93, 47.00, 39.02, 33.54, 26.03, 20.19. MS (ESI): *m*/*z* = 420.3 [M + H]^+^. Anal. Calcd. For C_25_H_29_N_3_O_3_: C, 71.57; H, 6.97; N, 10.02. Found: C, 71.43; H, 7.02; N, 10.17.

*(E)-3-(3,4-Dimethoxyphenyl)-N-(4-(6-methoxyquinolin-8-ylamino)pentyl)acrylamide* (**3d**): Compound **3d** was synthesized according to the general procedure using chloride **4d** (0.147 g, 0.65 mmol) and PQ diphosphate (0.228 g, 0.5 mmol) (method A) or compound **2d** (0.090 g, 0.29 mmol) and PQ diphosphate (0.132 g, 0.29 mmol) (method B). After purification by column chromatography (petroleum ether/EtOAc 1:2), pure compound **3d** was obtained (0.157 g, 70%, method A; 0.098 g, 75%; method B, m.p. = 85–88 °C). IR (KBr) (cm^−1^): 3355, 3256, 3072, 2964, 2932, 1738, 1653, 1613, 1555, 1518, 1458, 1425, 1388, 1339, 1298, 1259, 1201, 1166, 1138, 1022, 980, 851, 819, 793, 765, 681, 627, 597. ^1^H-NMR (δ*/*ppm): 8.54–8.53 (dd, 1H, *J* = 1.62, 4.19), 8.09–8.05 (dd, 1H *J* = 1.56, 8.29), 8.00–7.96 (t, 1H, *J* = 5.52), 7.44–7.40 (m, 1H, *J* = 4.20, 8.25), 7.36–7.31 (d, 1H, *J* = 15.73), 7.14–7.08 (m, 2H), 6.98–6.96 (d, 1H, *J* = 8.32), 6.51–6.45 (m, 2H), 6.28 (s, 1H), 6.15–6.12 (d, 1H, *J* = 8.76), 3.81–3.78 (m, 9H), 3.65 (m, 1H), 3.23–3.19 (m, 2H), 1.70–1.56 (m, 4H), 1.23–1.21 (d, 2H, *J* = 6.29). ^13^C-NMR (δ/ppm): 165.05, 158.97, 149.98, 148.85, 144.61, 144.18, 138.36, 134.73, 134.49, 129.53, 127.75, 122.03, 121.20, 120.05, 111.74, 109.96, 96.08, 91.61, 55.49, 55.37, 54.92, 47.00, 39.02, 33.55, 26.02, 20.20. MS (ESI): *m*/*z* = 450.5 [M + H]^+^. Anal. Calcd. For C_26_H_31_N_3_O_4_: C, 69.47; H, 6.95; N, 9.35. Found: C, 69.80; H, 7.01; N, 9.57.

*(E)-N-(4-(6-Methoxyquinolin-8-ylamino)pentyl)-3-(3,4,5-trimethoxyphenyl)acrylamide* (**3e**): Compound **3e** was synthesized according to the general procedure using chloride **4e** (0.169 g, 0.65 mmol) and PQ diphosphate (0.228 g, 0.5 mmol) (method A) or compound **2e** (0.109 g, 0.32 mmol) and PQ diphosphate (0.146 g, 0.32 mmol) (method B). After purification by column chromatography (petroleum ether/EtOAc/MeOH 30:10:5) and crystallization from EtOAc, pure compound **3e** was obtained (0.125 g, 52%, method A; 0.106 g, 69%, method B, m.p. = 120–123.5 °C). IR (KBr) (cm^−1^): 3367, 3285, 3084, 2962, 1657, 1617, 1583, 1520, 1456, 1421, 1388, 1328, 1277, 1241, 1221, 1200, 1164, 1125, 1052, 1014, 978, 824, 792, 682, 622, 604. ^1^H-NMR (δ/ppm): 8.54–8.53 (d, 1H, *J* = 2.85), 8.09–8.03 (m, 2H), 7.44–7.40 (m, 1H), 7.37–7.32 (d, 1H, *J* = 15.68), 6.88 (s, 2H), 6.54–6.52 (d, 1H, *J* = 15.69), 6.47 (s, 1H), 6.28 (s, 1H), 6.15–6.12 (d, 1H, *J* = 8.69), 3.81 (s, 9H), 3.68 (s, 4H), 3.21–3.19 (m, 2H), 1.69–1.65 (m, 4H), 1.23–1.22 (d, 3H, *J* = 6.15). ^13^C-NMR (δ/ppm): 164.83, 158.97, 153.00, 144.62, 144.18, 138.57, 138.46, 134.74, 134.49, 130.55, 129.53, 122.04, 121.66, 104.87, 96.08, 91.61, 60.02, 55.82, 54.92, 46.99, 39.02, 33.56, 25.98, 20.20. MS (ESI): *m*/*z* = 480.3 [M + H]^+^. Anal. Calcd. For C_27_H_33_N_3_O_5_: C, 67.62; H, 6.94; N, 8.76. Found: C, 67.99; H, 7.06; N, 8.99.

*(E)-3-(Benzo[d][1,3]dioxo-5-yl)-N-(4-(6-methoxyquinolin-8-ylamino)pentyl)acrylamide* (**3f**): Compound **3f** was synthesized according to the general procedure (method A) using chloride **4f** (0.137 g, 0.65 mmol) and PQ diphosphate (0.228 g, 0.5 mmol). After purification by column chromatography (CH_2_Cl_2_/MeOH = 97:3) and recrystallization from EtOAc/ether, pure compound **3f** was obtained (0.124 g, 57%, m.p. = 77–79.5 °C). IR (KBr) (cm^−1^): 3350, 3253, 3069, 2961, 2934, 1738, 1655, 1620, 1576, 1557, 1520, 1491, 1454, 1425, 1387, 1356, 1334, 1281, 1247, 1200, 1167, 1124, 1096, 1041, 977, 927, 855, 819, 792, 752, 681, 624, 593. ^1^H-NMR (δ/ppm): 8.53-8.51 (dd, 1H, *J* = 1.61, 4.18), 8.07–8.04 (dd, 1H, *J* = 1.57, 8.29), 7.99–7.95 (t, 1H, *J* = 5.52), 7.43–7.39 (m, 1H), 7.33–7.28 (d, 1H), 7.11 (s, 1H), 7.05–7.02 (dd, 1H, *J* = 1.46, 8.11), 6.93–6.91 (d, 1H, *J* = 8.00), 6.46–6.40 (m, 2H), 6.27 (s, 1H), 6.14–6.11 (d, 1H, *J* = 8.77), 6.04 (s, 2H), 3.80 (s, 3H), 3.64 (m, 1H), 3.21–3.15 (m, 2H), 1.69–1.51 (m, 4H), 1.22–1.20 (d, 2H, *J* = 6.29). ^13^C-NMR (δ*/* ppm): 164.93, 158.97, 148.29, 147.85, 144.61, 144.18, 138.13, 134.73, 134.49, 129.53, 129.33, 123.00, 122.05, 120.42, 108.49, 106.11, 101.33, 96.08, 91.61, 54.93, 46.99, 39.02, 33.53, 26.01, 20.19. MS (ESI): *m*/*z* = 434.2 [M + H]^+^. Anal. Calcd. For C_25_H_27_N_3_O_4_: C, 69.27; H, 6.28; N, 9.69. Found: C, 69.40; H, 6.69; N, 9.22.

*(E)-3-(4-Chlorophenyl)-N-(4-(6-methoxyquinolin-8-ylamino)pentyl)acrylamide* (**3g**): Compound **3g** was synthesized according to the general procedure using chloride **4g** (0.131 g, 0.65 mmol) and PQ diphosphate (0.228 g, 0.5 mmol) (method A) or **2g** (0.113 g, 0.4 mmol) and PQ diphosphate (0.182 g, 0.4 mmol) (method B). After purification by column chromatography (CH_2_Cl_2_/MeOH = 97:3), pure compound **3g** was obtained (0.167 g, 79%, method A; 0.056 g, 33%, method B, m.p. = 85–87 °C). IR (KBr) (cm^−1^): 3358, 3275, 3078, 2958, 2327, 2857, 1740, 1655, 1615, 1558, 1520, 1492, 1457, 1426, 1388, 1343, 1227, 1202, 1168, 1097, 1052, 1013, 982, 903, 823, 791, 736, 709, 677, 628. ^1^H-NMR (δ/ppm): 8.54–8.53 (dd, 1H, *J* = 1.60, 4.19), 8.13–8.05 (m, 2H), 7.58–7.56 (d, 2H, *J* = 8.54), 7.47–7.37 (m, 4H), 7.63–7.58 (d, 1H, *J* = 15.82), 7.47 (s, 1H), 6.28 (s, 1H), 6.15–6.12 (d, 1H, *J* = 8.79), 3.81 (s, 3H), 3.65 (m, 1H), 3.22–3.20 (m, 2H), 1.72–1.53 (m, 4H), 1.23–1.21 (d, 3H, *J* = 6.28). ^13^C-NMR (δ*/* ppm): 164.56, 158.97, 144.60, 144.18, 136.97, 134.73, 134.49, 133.91, 133.68, 129.53, 129.08), 128.85, 123.14, 122.03, 96.08, 91.61, 54.92, 46.98, 39.02, 33.51, 26.95, 20.19. MS (ESI): *m*/*z* = 424.2 [M + H]^+^. Anal. Calcd. For C_24_H_26_ClN_3_O_2_: C, 68.00; H, 6.18; N, 9.91. Found: C, 68.44; H, 6.35; N, 10.05.

*(E)-3-(2-Fluorophenyl)-N-(4-(6-methoxyquinolin-8-ylamino)pentyl)acrylamide* (**3h**): Compound **3h** was synthesized according to the general procedure (method A) using chloride **4h** (0.120 g, 0.65 mmol) and PQ diphosphate (0.228 g, 0.5 mmol). After purification by column chromatography (CH_2_Cl_2_/MeOH = 97:3) and recrystallization from EtOAc/ether, pure compound **3h** was obtained (0.120 g, 59%, m.p. = 65–67.5 °C). IR (KBr) (cm^−1^): 3357, 3271, 3078, 2960, 2927, 2857, 1739, 1655, 1616, 1578, 1557, 1520, 1487, 1458, 1426, 1388, 1343, 1283, 1226, 1201, 1167, 1093, 1052, 1032, 985, 903, 875, 822, 792, 758, 714, 678, 625. ^1^H-NMR (δ/ppm): 8.55–8.53 (dd, 1H, *J* = 1.60, 4.19), 8.23–8.19 (t, 1H, *J* = 5.50), 8.09–8.05 (dd, 1H, *J* = 1.55, 8.28), 7.66–7.61 (t, 1H, *J* = 6.49), 7.50–7.39 (m, 3H), 7.30–7.23 (m, 2H), 6.73–6.68 (d, 1H, *J* = 15.94), 6.47 (s, 1H), 6.28 (s, 1H), 6.15–6.13 (d, 1H, *J* = 8.79), 3.81 (s, 3H), 3.65 (m, 1H), 3.25–3.21 (m, 2H), 1.73–1.53 (m, 4H), 1.24–1.21 (d, 3H, *J* = 6.28). ^13^C-NMR (δ/ppm): 164.48, 162.02–158.71 (d, *J* = 250.43), 158.97, 144.61, 144.18, 134.73, 134.49, 131.14–131.02 (d, *J* = 8.58), 130.72, 129.53, 129.03–128.99 (d, *J* = 3.26), 125.17–125.09 (d, *J* = 6.18), 124.97, 122.61–122.41 (d, *J* = 12.66), 122.03, 116.12–115.83 (d, *J* = 23.47), 96.08, 91.61, 54.91, 46.98, 39.02, 33.51, 25.91, 20.19. MS (ESI): *m*/*z* = 408.1 [M + H]^+^. Anal. Calcd. For C_24_H_26_FN_3_O_2_: C, 70.74; H, 6.43; N, 10.31. Found: C, 70.60; H, 5.99; N, 10.39.

*(E)-N-(4-(6-Methoxyquinolin-8-ylamino)pentyl)-3-(3-trifluoromethyl)phenyl)acrylamide* (**3i**): Compound **3i** was synthesized according to the general procedure (method A) using chloride **4i** (0.152 g, 0.65 mmol) and PQ diphosphate (0.228 g, 0.5 mmol). After purification by column chromatography (CH_2_Cl_2_/MeOH = 97:3) and recrystallization from EtOAc/petroleum ether, pure compound **3i** was obtained (0.162 g, 71%, m.p. = 48.5–49.5 °C). IR (KBr) (cm^−1^): 3262, 3075, 2964, 2861, 1661, 1618, 1561, 1520, 1454, 1387, 1335, 1221, 1166, 1126, 975, 803, 864, 820, 793, 735, 688, 625, 584, 513. ^1^H-NMR (δ/ppm): 8.55–8.53 (dd, 1H, *J* = 1.46, 4.10), 8.15–8.11 (t, 1H, *J* = 5.51), 8.08–8.05 (dd, 1H, *J* = 1.56, 8.27), 7.89–7.84 (m, 2H), 7.73–7.62 (m, 2H), 7.52–7.47 (d, 1H, *J* = 15.84), 7.44–7.40 (m, 1H), 6.77–6.72 (d, 1H, *J* = 15.85), 6.47 (s, 1H), 6.29 (s, 1H), 6.16–6.13 (d, 1H, *J* = 8.76), 3.81 (s, 3H), 3.66 (m, 1H), 3.23–3.21 (m, 2H), 1.72–1.57 (m, 4H), 1.24–1.22 (d, 3H, *J* = 6.25). ^13^C-NMR (δ/ppm): 164.34, 158.97, 144.61, 144.18, 136.62, 136.17, 134.73, 134.50, 132.28–122.55 (q, *J* = 244.60), 131.20, 130.28–128.83 (q, *J* = 31.73), 129.96, 129.44, 125.60–125.46 (q, *J* = 3.70), 124.48, 123.75–123.60 (q, *J* = 3.71), 122.03, 96.09, 91.60, 54.91, 46.98, 39.02, 33.50, 25.91, 20.20. MS (ESI): *m*/*z* = 458.3 [M + H]^+^. Anal. Calcd. For C_25_H_26_F_3_N_3_O_2_: C, 65.63; H, 5.73; N, 9.18. Found: C, 65.24; H, 6.00; N, 9.43.

*(E)-N-(4-(6-Methoxyquinolin-8-ylamino)pentyl)-3-(4-(trifluoromethyl)phenyl)acrylamide* (**3j**): Compound **3j** was synthesized according to the general procedure (method A) using chloride **4j** (0.152 g, 0.65 mmol) and PQ diphosphate (0.228 g, 0.5 mmol). After purification by column chromatography (CH_2_Cl_2_/MeOH = 95:5), pure compound **3j** was obtained (0.167 g, 73%, m.p. = 82–84 °C). IR (KBr) (cm^−1^): 3359, 3262, 3075, 2963, 2931, 2856, 1738, 1655, 1615, 1577, 1560, 1519, 1456, 1424, 1386, 1326, 1227, 1203, 1163, 1130, 1110, 1069, 1050, 1015, 979, 957, 903, 880, 829, 792, 719, 681, 623, 596. ^1^H-NMR (δ/ppm): 8.55–8.53 (dd, 1H, *J* = 1.61, 4.19), 8.24–8.21 (t, 1H, *J* = 5.56), 8.09–8.06 (dd, 1H, *J* = 1.57, 8.30), 7.76 (s, 4H), 7.50–7.45 (d, 1H, *J* = 15.87), 7.45–7.40 (m, 1H), 6.76–6.71 (d, 1H, *J* = 15.84), 6.47 (s, 1H), 6.29 (s, 1H), 6.16–6.13 (d, 1H, *J* = 8.80), 3.81 (s, 3H), 3.66 (m, 1H), 3.25–3.21 (m, 2H), 1.73–1.53 (m, 4H), 1.24–1.21 (d, 3H, *J* = 6.28). ^13^C-NMR (δ/ppm): 164.31, 159.00, 144.63, 144.23, 139.07, 136.74, 134.79, 134.52, 129.57, 129.71–128.44 (q, *J* = 31.56), 129.51–118.69 (q, *J* = 272.14), 128.07, 125.82–125.67 (q, *J* = 3.80), 125.12, 122.09, 96.12, 91.60, 54.95, 46.99, 39.02, 33.52, 23.96, 20.23. MS (ESI): *m*/*z* = 458.2 [M + H]^+^. Anal. Calcd. For C_25_H_26_F_3_N_3_O_2_: C, 65.63; H, 5.73; N, 9.18. Found: C, 65.98; H, 6.01; N, 9.44.

*(E)-3-(3,5-Bis(trifluoromethyl)phenyl)-N-[4-(6-methoxyquinolin-8-ylamino)pentyl]acrylamide* (**3k**): Compound **3k** was synthesized according to the general procedure (method A) using chloride **4k** (0.097 g, 0.32 mmol) and PQ diphosphate (0.114 g, 0.25 mmol). After purification by column chromatography (CH_2_Cl_2_/MeOH = 95:5) and crystallization from ether, pure compound **3k** was obtained (0.097 g, 74%, m.p. = 148–150 °C). IR (KBr) (cm^−1^): 3395, 3286, 3095, 2964, 2934, 2861, 1663, 1623, 1576, 1521, 1456, 1423, 1334, 1341, 1278, 1224, 1174, 1136, 1052, 1031, 978, 940, 899, 868, 845, 822, 792, 683, 624. ^1^H-NMR (δ/ppm): 8.54 (s, 1H), 8.26 (s, 2H), 8.18 (t, 1H, *J* = 5.21), 8.08–8.07 (m, 2H), 7.60–7.58 (d, 1H, *J* = 15.82), 7.44–7.42 (m, 1H), 6.91–6.88 (d, 1H, *J* = 15.90), 6.47 (s, 1H), 6.28 (s, 1H), 6.16–6.14 (d, 1H, *J* = 8.54), 3.80 (s, 3H), 3.66 (m, 1H), 3.23 (m, 2H), 1.70–1.58 (m, 4H), 1.23–1.22 (d, 3H, *J* = 5.99). ^13^C-NMR (δ/ppm): 164.07, 159.00, 144.64, 144.24, 137.97, 135.16, 134.81, 134.53, 131.14–130.48 (q, *J* = 33.83), 129.58, 127.84, 126.61, 125.94–120.54 (q, *J* = 272.30), 122.23, 122.11, 96.14, 91.59, 54.95, 46.99, 39.02, 33.51, 25.92, 20.25. MS (ESI): *m*/*z* = 526.4 [M + H]^+^. Anal. Calcd. For C_26_H_25_F_6_N_3_O_2_: C, 59.43; H, 4.80; N, 8.00. Found: C, 59.67; H, 5.02; N, 8.22.

#### 3.1.5. Synthesis of *N*-(4-((6-Methoxyquinolin-8-yl)amino)pentyl)-1*H*-benzo[*d*][1,2,3]triazole-1-carboxamide (**5**)

Compound **5** was prepared according to our procedures published earlier [43].

#### 3.1.6. Synthesis of *N*-(4-((6-Methoxyquinolin-8-yl)amino)pentyl)hydrazinecarboxamide (**6**)

The title compound was prepared following previously published procedure [46].

#### 3.1.7. General Procedure for the Synthesis of Acylsemicarbazides **7a**–**k**

To a solution of chloride **4a**–**k** (1.1 equiv.) in dry dichloromethane, a solution of semicarbazide **6** (1 equiv.) and TEA (1 equiv.) in dry dichloromethane was added dropwise. The reaction mixture was stirred overnight at room temperature, light protected. The solvent was removed under reduced pressure and the residue was dissolved in ethyl acetate/5% NaOH mixture (1:1). The organic layer was extracted with 5% NaOH solution and water, dried over anhydrous sodium sulfate, filtered and evaporated. The crude product was purified by column chromatography.

*(E)-1-Cinnamoyl-4-(4-(6-methoxyquinolin-8-ylamino)pentyl)semicarbazide* (**7a**): Compound **7a** was synthesized according to the general procedure using chloride **4a** (0.183 g, 1.1 mmol) and compound **6** (0.317 g, 1 mmol). After purification by column chromatography (CH_2_Cl_2_/MeOH = 95:5) and crystallization from ether/petroleum ether, pure compound **7a** was obtained (0.251 g, 56%, m.p. = 188–190 °C, decomp.). IR (KBr) (cm^−1^): 3302, 3236, 3038, 2936, 1696, 1628, 1578, 1596, 1458, 1388, 1424, 1356, 1222, 1156, 1054, 978, 862, 822, 790, 762, 726, 678, 658, 632, 556, 488. ^1^H-NMR (δ/ppm): 9.75 (s, 1H), 8.54–8.53 (dd, 1H, *J* = 1.41), 8.09–8.06 (dd, 1H, *J* = 1.30, 8.25), 7.85 (s, 1H), 7.58–7.36 (m, 7H), 6.65–6.60 (d, 1H, *J* = 15.90), 6.48–6.44 (m, 2H), 6.28 (s, 1H), 6.13–6.10 (d, 1H, *J* = 8.62), 3.82 (s, 3H), 3.64 (m, 1H), 3.06–3.04 (m, 2H), 1.63–1.47 (m, 4H), 1.22–1.20 (d, 3H, *J* = 6.24). ^13^C-NMR (δ/ppm): 164.65, 158.98, 157.92, 144.61, 144.20, 139.46, 134.74, 134.67, 134.49, 129.60, 129.53, 128.92, 127.50, 122.04, 119.94, 96.07, 91.61, 54.95, 47.01, 39.02, 33.41, 26.65, 20.18. MS (ESI): *m*/*z* = 448.3 [M + H]^+^. Anal. Calcd. For C_25_H_29_N_5_O_3_: C, 67.09; H, 6.53; N, 15.65. Found: C, 67.37; H, 6.22; N, 15.66.

*(E)-4-(4-(6-Methoxyquinolin-8-ylamino)pentyl)-1-(2-methyl-3-phenylacryloyl)semicarbazide* (**7b**): Compound **7b** was synthesized according to the general procedure using chloride **4b** (0.197 g, 1.1 mmol) and compound **6** (0.317 g, 1 mmol). After purification by column chromatography (CH_2_Cl_2_/MeOH = 95:5), oily product **7b** was obtained, which crystallized after a long storage at low temperature (0.245 g, 53%, m.p. = 65.5 °C, decomp.). IR (KBr) (cm^−1^): 3265, 2961, 2935, 1652, 1617, 1576, 1520, 1454, 1423, 1387, 1336, 1258, 1239, 1220, 1203, 1158, 1051, 1031, 1004, 928, 910, 822, 791, 762, 709, 695, 625, 589, 515. ^1^H-NMR (δ/ppm): 9.67 (s, 1H, 2′′), 8.54–8.53 (dd, 1H, *J* = 1.57, 4.15), 8.08–8.07 (dd, 1H, *J* = 1.49, 8.28), 7.67 (s, 1H), 7.43–7.30 (m, 7H), 6.48–6.45 (m, 2H), 6.28 (s, 1H), 6.13–6.11 (d, 1H, *J* = 8.75), 4.05–3.82 (s, 3H), 3.65–3.63 (m, 1H), 3.08–3.02 (m, 2H), 2.02 (s, 3H), 1.66–1.47 (m, 4H), 1.22–1.21 (d, 3H, *J* = 6.29). ^13^C-NMR (δ/ppm): 168.89, 159.00, 158.36, 144.63, 144.24, 135.83, 134.78, 134.51, 133.23, 130.74, 129.56, 129.22, 128.42, 127.80, 122.08, 96.10, 91.61, 54.97, 47.02, 39.02, 33.43, 26.73, 20.21, 14.15. MS (ESI): *m*/*z* = 462.3 [M + H]^+^. Anal. Calcd. For C_26_H_31_N_5_O_3_: C, 67.66; H, 6.77; N, 15.17. Found: C, 67.51; H, 6.55; N, 15.00.

*(E)-1-(3-(4-Methoxyphenyl)acryloyl)-4-(4-(6-methoxyquinolin-8-ylamino)pentyl)semicarbazide* (**7c**): Compound **7c** was synthesized according to the general procedure using chloride **4c** (0.177 g, 0.9 mmol) and compound **6** (0.254 g, 0.8 mmol). After two purifications by column chromatography (first eluent: petroleum ether/EtOAc/MeOH = 30:10:5, second eluent CH_2_Cl_2_/MeOH = 95:5), pure compound **7c** was obtained (0.229 g, 60%, m.p. = 89.5 °C, decomp.). IR (KBr) (cm^−1^): 3248, 2934, 1654, 1604, 1575, 1517, 1456, 1424, 1386, 1251, 1167, 1029, 980, 824, 791, 628, 521. ^1^H-NMR (δ/ppm): 9.64 (s, 1H), 8.54–8.52 (dd, 1H, *J* = 1.45, 4.10), 8.08–8.06 (d, 1H, *J* = 7.07), 7.80 (s, 1H), 7.53–7.42 (m, 4H), 6.99–6.97 (d, 2H, *J* = 8.66), 6.49–6.45 (m, 3H), 6.27 (s, 1H), 6.10 (s, 1H), 3.82 (s, 3H), 3.78 (s, 3H), 3.62 (m, 1H), 3.04 (m, 2H), 1.63–1.46 (m, 4H), 1.21–1.19 (d, 3H, *J* = 6.23). ^13^C-NMR (δ/ppm): 165.24, 160.56, 159.06, 158.17, 144.69, 144.35, 139.41, 134.90, 134.56, 129.65 (14), 129.28 (4′, 8′), 127.30 (3′), 122.19 (12), 117.37 (1′), 114.49 (5′, 7′), 96.19 (17), 91.71, 55.34, 55.07, 47.11, 39.02, 33.49, 26.77, 20.29. MS (ESI): *m*/*z* = 478.3 [M + H]^+^. Anal. Calcd. For C_26_H_31_N_5_O_4_: C, 65.39; H, 6.54; N, 14.66. Found: C, 65.51; H, 6.60; N, 14.48.

*(E)-1-(3-(3,4-Dimethoxyphenyl)acryloyl)-4-(4-(6-methoxyquinolin-8-ylamino)pentyl)semicarbazide* (**7d**): Compound **7d** was synthesized according to the general procedure using chloride **4d** (0.227 g, 1 mmol) and compound **6** (0.286 g, 0.9 mmol). After trituration with ethyl acetate, dichloromethane, hot acetone, ether and hot ethanol, pure compound **7d** was obtained (0.260 g, 57%, m.p. = 200.5–201.5 °C, decomp.). IR (KBr) (cm^−1^): 3375, 3213, 3084, 3003, 2937, 1668, 1628, 1596, 1559, 1515, 1456, 1421, 1387, 1356, 1293, 1262, 1235, 1202, 1171, 1138, 1052, 1022, 977, 939, 856, 816, 788, 767, 710, 679, 622, 594, 560, 460. ^1^H-NMR (δ/ppm): 9.62–9.61 (d, 1H, *J* = 1.73), 8.54–8.52 (dd, 1H, *J* = 1.39, 4.06), 8.08–8.06 (dd, 1H, *J* = 1.29, 8.29), 7.81 (s, 1H), 7.44–7.40 (m, 2H), 7.15–7.12 (m, 2H), 7.00–6.98 (d, 1H, *J* = 8.28), 6.53–6.46 (m, 3H), 6.26 (s, 1H), 6.11–6.09 (d, 1H, *J* = 8.73), 3.81–3.78 (m, 9H), 3.62 (m, 1H), 3.04–3.02 (m, 2H), 1.54–1.51 (m, 4H), 1.21–1.19 (d, 3H, *J* = 6.25). ^13^C-NMR (δ/ppm): 165.27, 159.06, 158.19, 150.32, 148.93, 144.69, 144.36, 139.74, 134.91, 134.57, 129.66, 127.55, 122.21, 121.48, 117.64, 111.81, 110.20, 96.19, 91.69, 55.62, 55.52, 55.08, 47.11, 39.02, 33.50, 26.80, 20.30. MS (ESI): *m*/*z* = 508.3 [M + H]^+^. Anal. Calcd. For C_27_H_33_N_5_O_5_: C, 63.89; H, 6.55; N, 13.80. Found: C, 64.03; H, 6.68; N, 13.50.

*(E)-4-(4-(6-Methoxyquinolin-8-ylamino)pentyl)-1-(3-(3,4,5-trimethoxyphenyl)acryloyl)semicarbazide* (**7e**): Compound **7e** was synthesized according to the general procedure using chloride **4e** (0.257 g, 1 mmol) and compound **6** (0.286 g, 0.9 mmol). After purification by column chromatography (CH_2_Cl_2_/MeOH = 95:5) and crystallization from ether/petroleum ether, pure compound **7e** was isolated (0.300 g, 62%, m.p. = 90.5 °C, decomp.). IR (KBr) (cm^−1^): 3250, 2937, 1654, 1618, 1582, 1508, 1454, 1420, 1388, 1326, 1266, 1240, 1221, 1203, 1155, 1126, 1051, 1031, 1003, 976, 822, 791, 677, 624, 585, 526, 512, 464. ^1^H-NMR (δ/ppm): 9.64–9.63 (d, 1H, *J* = 1.91), 8.53–8.52 (dd, 1H, *J* = 1.51, 4.16), 8.07–8.05 (dd, 1H, *J* = 1.41, 8.28), 7.83 (s, 1H), 7.45–7.40 (m, 2H), 6.90 (s, 2H), 6.59–6.55 (d, 1H, *J* = 15.80), 6.46–6.43 (m, 2H), 6.26 (s, 1H), 6.11–6.09 (d, 1H, *J* = 8.78), 3.81 (s, 3H), 3.80 (s, 6H), 3.68 (s, 3H), 3.63 (m, 1H), 3.04–3.02 (m, 2H), 1.61–1.51 (m, 4H), 1.21–1.19 (d, 3H, *J* = 6.25). ^13^C-NMR (δ/ppm): 164.92, 159.05, 158.05, 153.14, 144.69, 144.33, 139.82, 138.89, 134.88, 134.56, 130.38, 129.64, 122.17, 119.26, 105.09, 96.17, 91.69, 60.16, 55.96, 55.05, 47.10, 39.02, 33.49, 26.76, 20.28. MS (ESI): *m*/*z* = 538.3 [M + H]^+^. Anal. Calcd. For C_28_H_35_N_5_O_6_: C, 62.55; H, 6.56; N, 13.03. Found: C, 62.79; H, 6.86; N, 13.29.

*(E)-1-(3-(Benzo[d][1,3]dioxo-5-yl)acryloyl)-4-(4-(6-methoxyquinolin-8-ylamino)pentyl)semicarbazide* (**7f**): Compound **7f** was synthesized according to the general procedure using chloride **4f** (0.211 g, 1 mmol) and compound **6** (0.286 g, 0.9 mmol). After purification by column chromatography (CH_2_Cl_2_/MeOH = 95:5) and crystallization from ether/petroleum ether, pure compound **7f** was obtained (0.217 g, 49%, m.p. = 75.5 °C, decomp.). IR (KBr) (cm^−1^): 3250, 2335, 2361, 1654, 1618, 1577, 1560, 1521, 1490, 1448, 1388, 1252, 1202, 1158, 1100, 1037, 976, 929, 819, 791, 670, 625, 592, 518. ^1^H-NMR (δ/ppm): 9.64 (s, 1H), 8.53 (d, 1H, *J* = 2.84), 8.08–8.06 (d, 1H, *J* = 8.05), 7.82 (s, 1H), 7.43–7.39 (m, 2H), 7.15 (s, 1H), 7.09–7.07 (d, 1H, *J* = 8.05), 6.96–6.94 (d, 1H, *J* = 7.96), 6.48–6.44 (m, 3H), 6.26 (s, 1H), 6.11–6.09 (d, 1H, *J* = 7.59), 6.06 (s, 3H), 3.82 (s, 3H), 3.62 (m, 1H), 3.04 (m, 2H), 1.60–1.46 (m, 4H), 1.12–1.19 (d, 3H, *J* = 6.13). ^13^C-NMR (δ/ppm): 165.45, 159.43, 158.49, 149.08, 148.38, 145.05, 144.69, 139.84, 135.25, 134.93, 130.01, 129.50, 123.83, 122.54, 118.33, 109.05, 106.66, 101.92, 96.55, 92.06, 55.42, 47.47, 39.02, 33.86, 27.14, 20.65. MS (ESI): *m*/*z* = 492.3 [M + H]^+^. Anal. Calcd. For C_26_H_29_N_5_O_5_: C, 63.53; H, 5.95; N, 14.25. Found: C, 63.73; H, 5.84; N, 14.00.

*(E)-1-(3-(4-Chlorophenyl)acryloyl)-4-(4-(6-methoxyquinolin-8-ylamino)pentyl)semicarbazide* (**7g**): Compound **7g** was synthesized according to the general procedure using chloride **4g** (0.201 g, 1 mmol) and compound **6** (0.286 g, 0.9 mmol). After purification by column chromatography (petroleum ether/EtOAc/MeOH = 30:10:5) and trituration with ether, compound **7g** was obtained (0.256 g, 59%, m.p. = 185–187.5 °C). IR (KBr) (cm^−1^): 3337, 3230, 3037, 2966, 2937, 2362, 2343, 1697, 1661, 1625, 1592, 1521, 1490, 1458, 1425, 1408, 1391, 1354, 1290, 1266, 1239, 1221, 1199, 1161, 1090, 1052, 1010, 982, 945, 899, 866, 819, 790, 726, 676, 628, 495. ^1^H-NMR (δ/ppm): 9.79 (s, 1H), 8.53–8.52 (dd, 1H, *J* = 1.06, 4.11), 8.08–8.05 (dd, 1H, *J* = 1.30, 8.25), 7.88 (s, 1H), 7.61–7.59 (d, 2H, *J* = 8.47), 7.50–7.45 (m, 3H), 7.43–7.40 (m, 1H), 6.64–6.60 (d, 1H, *J* = 15.89), 6.51–6.48 (t, 1H, *J* = 5.44), 6.46 (s, 1H), 6.25 (s, 1H), 6.11–6.09 (d, 1H, *J* = 8.71), 3.81 (s, 3H), 3.62 (m, 1H), 3.04 (m, 2H), 1.61–1.51 (m, 4H), 1.20–1.19 (d, 3H, *J* = 6.25). ^13^C-NMR (δ/ppm): 164.63, 159.05, 158.03, 144.68, 144.33, 138.28, 134.89, 134.56, 134.18, 133.68, 129.64, 129.35, 129.09, 122.19, 120.75, 96.17, 91.66, 55.06, 47.08, 39.02, 33.47, 26.77, 20.28. MS (ESI): *m*/*z* = 482.2 [M + H]^+^. Anal. Calcd. For C_25_H_28_ClN_5_O_3_: C, 62.30; H, 5.86; N, 14.53. Found: C, 62.69; H, 6.01; N, 14.30.

*(E)-1-(3-(2-Fluorophenyl)acryloyl)-4-(4-(6-methoxyquinolin-8-ylamino)pentyl)semicarbazide* (**7h**): Compound **7h** was synthesized according to the general procedure using chloride **4h** (0.185 g, 1 mmol) and compound **6** (0.286 g, 0.9 mmol). After purification by column chromatography (CH_2_Cl_2_/MeOH = 95:5) and trituration with ether, pure compound **7h** was obtained (0.372 g, 89%, m.p. = 91–93 °C, decomp.). IR (KBr) (cm^−1^): 3250, 2964, 1654, 1618, 1578, 1520, 1457, 1388, 1221, 1159, 1052, 982, 822, 791, 757. ^1^H-NMR (δ/ppm): 9.85 (s, 1H), 8.54–8.53 (dd, 1H, *J* = 1.50, 4.10), 8.08-8.06 (dd, 1H, *J* = 1.42, 8.22), 7.87 (s, 1H), 7.67–7.65 (t, 1H, *J* = 7.22), 7.57–7.55 (d, 1H, *J* = 16.02), 7.45–7.41 (m, 2H), 7.30–7.26 (m, 2H), 6.76–6.73 (d, 1H, *J* = 16.03), 6.48 (s, 2H), 6.28 (s, 1H), 6.12–6.11 (d, 1H, *J* = 8.70), 3.83 (s, 3H), 3.64 (m, 1H), 3.07 (m, 2H), 1.65–1.49 (m, 4H), 1.22–1.21 (d, 3H, *J* = 6.28). ^13^C-NMR (δ/ppm): 164.43, 161.30–159.64 (d, *J* = 250.54), 158.99, 157.86, 144.62, 144.21), 134.74, 134.50, 131.90, 131.47–131.41 (d, *J* = 8.67), 129.54, 129.22–129.20 (d, *J* = 2.69), 124.98, 122.90–122.86 (d, *J* = 6.35), 122.35–122.28 (d, *J* = 11.54), 122.04, 116.13–115.99 (d, *J* = 21.68), 96.09, 91.04, 54.95, 47.03, 39.02, 33.43, 26.64, 20.19. MS (ESI): *m*/*z* = 466.1 [M + H]^+^. Anal. Calcd. For C_25_H_28_FN_5_O_3_: C, 64.50; H, 6.06; N, 15.04. Found: C, 64.69; H, 6.17; N, 15.33.

*(E)-4-(4-(6-Methoxyquinolin-8-ylamino)pentyl)-1-(3-(3-(trifluoromethyl)phenyl)acryloyl)semicarbazide* (**7i**): Compound **7i** was synthesized according to the general procedure using chloride **4i** (0.235 g, 1 mmol) and compound **6** (0.286 g, 0.9 mmol). After purification by column chromatography (CH_2_Cl_2_/MeOH = 95:5) and crystallization from dichloromethane, pure compound **7i** was obtained (0.343 g, 74%, m.p. = 86–88 °C, decomp. IR (KBr) (cm^−1^): 3254, 2964, 1659, 1618, 1577, 1521, 1456, 1424, 1388, 1334, 1222, 1199, 1166, 1126, 1075, 976, 900, 822, 792, 694, 660, 625, 561, 514. ^1^H-NMR (δ/ppm): 9.77 (s, 1H), 8.54 (d, 1H, *J* = 2.58), 8.08–8.06 (d, 1H, *J* = 7.91), 7.92–7.88 (m, 3H), 7.75–7.73 (d, 1H, *J* = 7.53), 7.68–7.65 (t, 1H, *J* = 7.55), 7.61–7.59 (d, 1H, *J* = 15.88), 7.43–7.41 (m, 1H), 6.78–6.76 (d, 1H, *J* = 15.89), 6.47 (s, 2H), 6.28 (s, 1H), 6.12–6.11 (d, 1H, *J* = 8.50), 3.83 (s, 3H), 3.64 (m, 1H), 3.07–3.06 (m, 2H), 1.65–1.49 (m, 4H), 1.22–1.21 (d, 3H, *J* = 6.00). ^13^C-NMR (δ/ppm): 164.18, 158.99, 157.81, 144.62, 144.20, 137.75, 135.90, 134.74, 134.50, 132.14–127.87 (q, *J* = 214.50), 131.15, 130.07–129.41 (q, *J* = 30.02), 130.06, 129.53, 125.84, 123.98, 122.11, 122.03, 96.08, 91.64, 54.94, 47.03, 39.02, 33.43, 26.64, 20.18. MS (ESI): *m*/*z* = 516.1 [M + H]^+^. Anal. Calcd. For C_26_H_28_F_3_N_5_O_3_: C, 60.57; H, 5.47; N, 13.58. Found: C, 60.60; H, 5.81; N, 13.77.

*(E)-4-(4-(6-Methoxyquinolin-8-ylamino)pentyl)-1-(3-(4-(trifluoromethyl)phenyl)acryloyl)semicarbazide* (**7j**): Compound **7j** was synthesized according to the general procedure using chloride **4j** (0.235 g, 1 mmol) and compound **6** (0.286 g, 0.9 mmol). After purification by column chromatography (CH_2_Cl_2_/MeOH = 95:5) and crystallization from ether/petroleum ether, pure compound **7j** was obtained (m.p. = 123 °C, decomp.). A portion of product **7j** crystallized from the reaction mixture. It was filtered off and washed with water. Overall yield: 0.311 g, 67%. IR (KBr) (cm^−1^): 3266, 1695, 1664, 1612, 1525, 1469, 1425, 1390, 1327, 1235, 1169, 1118, 1067, 834, 789, 728, 631, 592. ^1^H-NMR (δ/ ppm): 9.85 (s, 1H), 8.54–8.53 (d, 1H, *J* = 3.71), 8.08–8.06 (d, 1H, *J* = 8.07), 7.90 (s, 1H), 7.78 (s, 4H), 7.59–7.56 (d, 1H, *J* = 15.92), 7.43–7.41 (m, 1H), 6.77–6.74 (d, 1H, *J* = 15.90), 6.47 (s, 2H), 6.28 (s, 1H), 6.12–6.11 (d, 1H, *J* = 8.58), 3.83 (s, 3H), 3.64 (m, 1H), 3.07–3.05 (m, 2H), 1.65–1.47 (m, 4H), 1.22–1.21 (d, 3H, *J* = 6.06). ^13^C-NMR (δ/ ppm): 164.10, 158.98, 157.80, 144.61, 144.19, 138.73, 137.75, 134.73, 134.49, 129.64–129.01 (q, *J* = 31.19), 129.53, 128.13, 126.81–121.32 (q, *J* = 271.67), 125.76, 122.77, 122.02, 96.07, 91.63, 54.93, 47.01, 39.02, 33.42, 26.63, 20.17. MS (ESI): *m*/*z* = 516.2 [M + H]^+^. Anal. Calcd. For C_26_H_28_F_3_N_5_O_3_: C, 60.57; H, 5.47; N, 13.58. Found: C, 60.22; H, 5.24; N, 13.33.

*(E)-1-(3-(3,5-Bis(trifluoromethyl)phenyl)acryloyl)-4-(4-(6-methoxyquinolin-8-ylamino)pentyl)semicarbazide* (**7k**): Compound **7k** was synthesized according to the general procedure using chloride **4k** (0.303 g, 1 mmol) and compound **6** (0.286 g, 0.9 mmol). After purification by column chromatography (CH_2_Cl_2_/MeOH = 95:5) and crystallization from toluene, pure compound **7k** was obtained (0.294 g, 56%, m.p. = 118–119.5 °C, decomp.). IR (KBr) (cm^−1^): 3393, 3335, 3220, 3020, 2942, 2363, 1647, 1617, 1579, 1521, 1459, 1425, 1384, 1340, 1280, 1224, 1175, 1135, 1053, 971, 942, 898, 847, 823, 792, 730, 683, 629, 599, 561, 519, 466. ^1^H-NMR (δ/ppm): 9.81 (s, 1H), 8.55–8.53 (dd, 1H, *J* = 1.62, 4.19), 8.29 (s, 2H), 8.12 (s, 1H), 8.09–8.07 (dd, 1H, *J* = 1.58, 8.31), 7.99 (s, 1H), 7.72–7.68 (d, 1H, *J* = 15.92), 7.77–7.41 (m, 1H), 6.94–6.90 (d, 1H, *J* = 15.97), 6.51–6.47 (m, 2H), 6.28 (s, 1H), 6.14–6.11 (d, 1H, *J* = 8.74), 3.82 (s, 3H), 3.64 (m, 1H), 3.06 (m, 2H), 1.66–1.48 (m, 4H), 1.22–1.21 (d, 3H, *J* = 6.28). ^13^C-NMR (δ/ppm): 163.86, 159.02, 157.78, 144.65, 144.27, 137.67, 136.28, 134.83, 134.53, 131.40–130.40 (q, *J* = 33.53), 129.60, 128.22, 127.28–119.14 (q, *J* = 272.61), 125.53, 124.23, 122.13, 96.13, 91.61, 55.01, 47.04, 39.02, 33.45, 26.74, 20.25. MS (ESI): *m*/*z* = 584.3 [M + H]^+^. Anal. Calcd. For C_27_H_27_F_6_N_5_O_3_: C, 55.57; H, 4.66; N, 12.00. Found: C, 55.60; H, 4.83; N, 12.29.

### 3.2. Biological Evaluation

#### 3.2.1. Anticancer Activity

The experiments were carried out on a murine lymphocytic leukemia cell line (L1210) and five human tumor cell lines: CEM, HeLa, NCI-H460, SW 620 and MCF-7. The cells were cultured as monolayers and maintained in Dulbecco′s modified Eagle medium (DMEM) supplemented with 10% fetal bovine serum (FBS), 2 mM l-glutamine, 100 U/mL penicillin and 100 μg/mL streptomycin in a humidified atmosphere with 5% CO_2_ at 37 °C.

The growth inhibition activity of the compounds was assessed as described previously [46]. The cell lines were inoculated onto a series of standard 96-well microtiter plates on day 0, at 1.5 × 10^4^ cells/mL (SW 620, H460) to 2.5 × 10^4^ cells/mL (MCF-7, CEM and L1210), depending on the growth of the tumor cell line. Test agents were then added in ten-fold dilutions (10^−8^ to 10^−4^ M) and the cell cultures were then incubated for 3–4 days with 5% CO_2_ at 37 °C. Working dilutions of the compounds were freshly prepared on the day of testing. 

After 3 (MCF-7, SW 620, NCI-H460) or 4 days (CEM, L1210), the cell growth rate was evaluated first light microscopically, then by viable cell counting and/or by performing the MTT assay, which detects dehydrogenase activity in live, not dead, cells. The absorbance (*A*) was measured on a microplate reader at 570 nm. The absorbance is directly proportional to the number of living, metabolically active cells. The results are expressed as IC_50_, which is the concentration necessary for 50% of inhibition. The IC_50_ values for each compound are calculated from concentration-response curves using linear regression analysis by fitting the test concentrations that give percentage of growth (PG) values above and below the reference value (i.e., 50%). If however, all of the tested concentrations produce PGs exceeding the respective reference level of effect (e.g., PG value of 50), then the highest tested concentration is assigned as the default value, which is preceded by a “>” sign. Minimum two individual experiments were carried out and each test point was performed in quadruplicate.

#### 3.2.2. Antiviral Activity

Antiviral activity against herpes simplex virus type 1 (KOS), herpes simplex virus 2 (G), herpes simplex virus 1 TK^−^ (KOS) ACV^r^, vaccinia virus, adeno virus 2 and human coronavirus (229E) was determined essentially as described previously [57,58]. After a 2 h incubation period, residual virus was removed and the infected cells were further incubated with the medium containing different concentration of the test compounds. After incubation for 3 days at 37 °C, virus-induced cytopathogenicity was monitored microscopically. Antiviral activity was expressed as the concentration required to reduce virus-induced cytopathogenicity by 50% (EC_50_).

#### 3.2.3. Cytotoxicity Assays

Cytotoxicity measurements were based on the inhibition of HEL cell growth. Cells were seeded at 5 × 10^3^ cells/well into 96-well microtiter plates. Then, medium containing different concentrations of the test compounds was added. After 3 days of incubation at 37 °C, the alteration of morphology of the cell cultures was recorded light microscopically. Cytotoxicity was expressed as MCC.

#### 3.2.4. Interaction of the New Derivatives with the Stable Radical DPPH

To a solution of DPPH in absolute ethanol the appropriate volume of the compounds (50 or 100 µM final concentrations) dissolved in DMSO was added. The absorbance was recorded at 517 nm after 20 and 60 min at room temperature. The experiments were repeated at least in triplicate and the standard deviation of absorbance was less than 10% of the mean [47]. NDGA was used under the same experimental conditions as a reference compound.

#### 3.2.5. Inhibition of Linoleic Acid Peroxidation

For initiating the lipid peroxidation free radical AAPH was used [47]. Final solution in the UV cuvette consisted of 10 μL linoleate sodium solution (*c* = 16 mM), 0.93 mL phosphate buffer (*c* = 0.05 M), pH 7.4, thermostated at 37 °C. 50 μL AAPH solution (*c* = 40 mM) and 10 μL of the tested compounds were added. The experiment was performed at 37 °C under air. The oxidation of linoleic acid sodium salt was monitored at 234 nm. The assays were repeated at least in triplicate and the standard deviation of absorbance was less than 10% of the mean. Trolox was used under the same experimental conditions as a reference compound.

#### 3.2.6. Anti-inflammatory Activity: LOX Inhibition Study In Vitro

LOX inhibitory assay in vitro was accomplished as described previously [47]. The test compounds (stock solutions 10 mM in DMSO) were incubated at room temperature with sodium linoleate (*c* = 0.1 mM) and 0.2 mL of LOX enzyme solution (1/9 × 10^−4^ *m*/*V* in saline). The conversion of sodium linoleate to 13-hydroperoxylinoleic acid was measured at 234 nm and compared with the reference inhibitor. Several concentrations were used for the IC_50_ determination. The assays were repeated at least in triplicate and the standard deviation of absorbance was less than 10% of the mean. NDGA was used under the same experimental conditions as a reference compound.

## 4. Conclusions

We have designed and synthesized a new scaffold comprising CADs and PQ motifs bound by amide (compounds **3a**–**k**) or acylsemicarbazide bonds (compounds **7a**–**k**). Twenty two PQ-CAD conjugates were prepared and subjected to extensive biological evaluation. Anticancer screening in vitro against six selected cancer cell lines showed that acylsemicarbazide derivatives were, in general, more active than amide derivatives. Almost all compounds from series **3** were selective towards MCF-7 cell line and active in micromolar concentrations. The most selective compound from the amide series was the *o*-fluoro derivative **3h**. It showed high activity against HeLa, MCF-7 and in particular against the SW 620 cell line. Four acylsemicarbazide derivatives, namely **7f** with benzodioxole ring and **7c**, **7g** and especially **7j** with methoxy, chloro and trifluoromethyl substituents in *para* position of cinnamic benzene ring, showed high selectivity and very strong activity against MCF-7 cell line in micromolar (**7c**, **7f** and **7g**) and nanomolar levels (**7j**). These compounds might provide fertile and much needed leads in anticancer drug discovery. 

Trifluoromethyl acylsemicarbazide derivatives **7i**, **7j** and 7**k** showed selective antiviral activity against human coronavirus (strain 229E) at concentrations which did not alter normal cell morphology and could be, therefore, considered as potential drug leads for further structural optimization. The same compounds, together with **7d** and **7g**, exerted the most potent antioxidant abilities in interaction with the free stable radical DPPH, while unsubstituted (**7a**), methoxy (**7c**–**e**), benzodioxole (**7f**), *p*-Cl (**7g**) and *m*-CF_3_ (**7i**) acylsemicarbazides and amide **3f** presented the highest anti-lipid peroxidation activity (83–89%). Dimethoxy derivative **7d** was the most potent LOX inhibitor (IC_50_ = 10 μΜ). The above results support the principal idea of PQ-CADs hybridization leading to multifunctional agents.

## Figures and Tables

**Table 1:** Properties of the PQ-CAD conjugates calculated with Chemicalize.org program [54]. The Lipinski and Gelovani parameters.

Compd.	Molecular Formula	Number of Atoms	MW	log *P*	H-Bond Donor	H-Bond Acceptor	Lipinski Score ^a^	MR (cm^3^/mol)	PSA (Å^2^)
**3a**	C_24_H_27_N_3_O_2_	56	389.49	3.82	2	4	4	118.37	63.25
**3b**	C_25_H_29_N_3_O_2_	59	403.53	4.22	2	4	4	122.73	63.25
**3c**	C_25_H_29_N_3_O_3_	60	419.52	3.66	2	5	4	124.84	72.48
**3d**	C_26_H_31_N_3_O_4_	64	449.54	3.51	2	6	4	131.30 ^b^	81.71
**3e**	C_27_H_33_N_3_O_5_	68	479.57	3.35	2	7	4	137.76 ^b^	90.94
**3f**	C_25_H_27_N_3_O_4_	59	433.50	3.44	2	6	4	124.14	81.71
**3g**	C_24_H_26_ClN_3_O_2_	56	423.94	4.43	2	4	4	123.18	63.25
**3h**	C_24_H_26_FN_3_O_2_	56	407.48	3.96	2	4	4	118.59	63.25
**3i**	C_25_H_26_F_3_N_3_O_2_	59	457.49	4.70	2	4	4	124.35	63.25
**3j**	C_25_H_26_F_3_N_3_O_2_	59	457.49	4.70	2	4	4	124.35	63.25
**3k**	C_26_H_25_F_6_N_3_O_2_	62	525.49	5.58	2	4	2	130.32	63.25
**7a**	C_25_H_29_N_5_O_3_	62	447.53	2.90	4	5	4	129.92	104.38
**7b**	C_26_H_31_N_5_O_3_	65	461.56	3.29	4	5	4	134.27 ^b^	104.38
**7c**	C_26_H_31_N_5_O_4_	66	477.56	2.74	4	9	4	136.38 ^b^	113.61
**7d**	C_27_H_33_N_5_O_5_	70	507.58 ^b^	2.58	4	7	3 ^b^	142.84	122.84
**7e**	C_28_H_35_N_5_O_6_	74 ^b^	537.61	2.43	4	8	3	149.31	132.07
**7f**	C_26_H_29_N_5_O_5_	65	491.54	2.52	4	7	4	135.68 ^b^	122.84
**7g**	C_25_H_28_ClN_5_O_3_	62	481.97	3.50	4	8	4	134.72 ^b^	104.38
**7h**	C_25_H_28_FN_5_O_3_	62	465.52	3.04	4	5	4	130.13	104.38
**7i**	C_26_H_28_F_3_N_5_O_3_	65	515.53 ^b^	3.78	4	5	3 ^b^	135.89 ^b^	104.38
**7j**	C_26_H_28_F_3_N_5_O_3_	65	515.53 ^b^	3.78	4	5	3 ^b^	135.89 ^b^	104.38
**7k**	C_27_H_27_F_6_N_5_O_3_	68	583.53	4.65	4	5	3	141.86	104.38

^a^ out of four; ^b^ minimal aberrations of the rules; MR—molecular refractivity; PSA—polar surface area.

**Table 2:** Growth inhibition of tumor cell lines in vitro: IC_50_ (μM) ^a^.

Compd.	Structural Formula	Cell Line					
		L1210	CEM	HeLa	NCI-H460	SW 620	MCF-7
**3a**	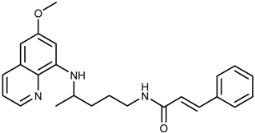	52 ± 3	27 ± 4	4.0 ± 0.9	>1	23 ± 9	24 ± 5
**3b**	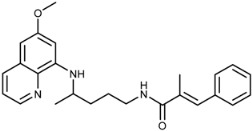	51 ± 0	55 ± 6	106 ± 26	>100	>100	9.4 ± 0.2
**3c**	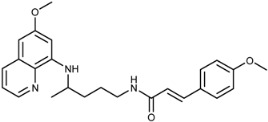	106 ± 6	61 ± 30	>125	>100	>100	20 ± 3
**3d**	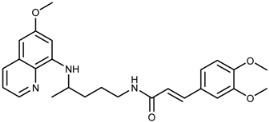	100 ± 34	90 ± 45	>125	>100	>100	16 ± 0.6
**3e**	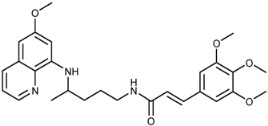	22 ± 2	55 ± 16	>125	>100	>100	8.7 ± 0.3
**3f**	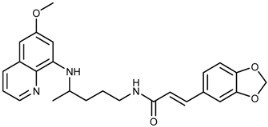	59 ± 2	37 ± 27	72 ± 57	>100	>100	6.9 ± 1.3
**3g**	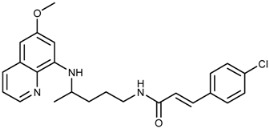	63 ± 2	20 ± 2	36 ± 16	>100	>100	4.3 ± 1.0
**3h**	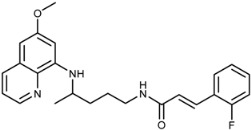	66 ± 5	18 ± 15	2.1 ± 2.1	>100	0.3 ± 0.1	1.1 ± 0.6
**3i**	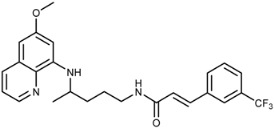	68 ± 2	41 ± 15	112 ± 11	>100	64 ± 41	11 ± 2
**3j**	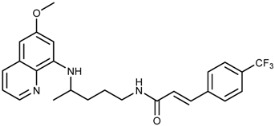	50 ± 18	14 ± 1	25 ± 6	>100	>100	3.9 ± 0.6
**3k**	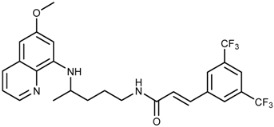	92 ± 24	68 ± 29	18 ± 1	>100	>100	2.6 ± 0.5
**7a**	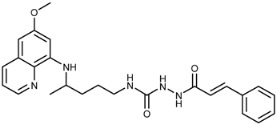	7.0 ± 3.1	3.0 ± 0.5	12 ± 2	>100	>100	2.5 ± 1.9
**7b**	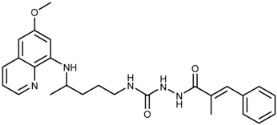	53 ± 5	25 ± 14	47 ± 6	50 ± 4	9.5 ± 0.9	16 ± 9
**7c**	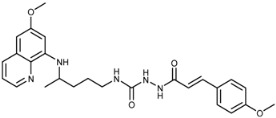	1.7 ± 0.4	1.3 ± 0.7	2.4 ± 0.2	>100	>100	0.4 ± 0.2
**7d**	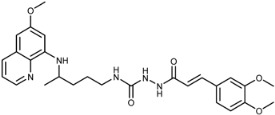	40 ± 14	93 ± 46	92 ± 30	32 ± 21	25 ± 9	1.9 ± 1.8
**7e**	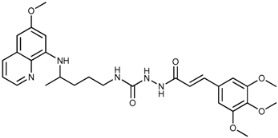	57 ± 5	54 ± 12	70 ± 2	63 ± 2	48 ± 17	1.4 ± 0.1
**7f**	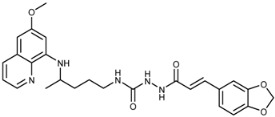	4.8 ± 0.2	7.4 ± 2.1	31 ± 0	27 ± 3	17 ± 5	0.6 ± 0.3
**7g**	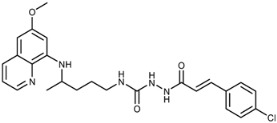	1.6 ± 0.7	0.9 ± 0.7	2.7 ± 1.2	20 ± 0.7	21 ± 4	0.2 ± 0.2
**7h**	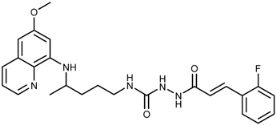	40 ± 4	15 ± 2	46 ± 2	12 ± 0.2	12 ± 4	5.9 ± 2.0
**7i**	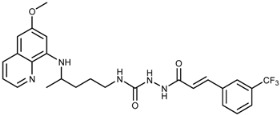	9.4 ± 4.8	10 ± 1	10 ± 1	18 ± 3	16 ± 5	2.3 ± 0.2
**7j**	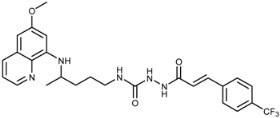	27 ± 0	9.4 ± 5.6	28 ± 12	34 ± 2	40 ± 5	0.03 ± 0.02
**7k**	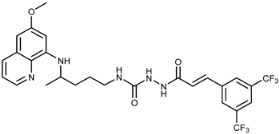	30 ± 17	17 ± 5	30 ± 23	48 ± 2	32 ± 4	3.2 ± 0.8
PQ		–	–	–	30 ± 7	20 ± 6 ^b^	28 ± 10
SOR		4.2 ± 2.4	3.2 ± 1.7	7.1 ± 2.6	6.1 ± 0.6 ^c^	7.1 ± 1.9	3.9 ± 1.6
CIS		–	–	–	1 ± 0.1	7 ± 2 ^b^	10 ± 1
5-FU		0.5 ± 0.2	18 ± 5	0.54 ± 0.1	3 ± 0.3	4 ± 0.7 ^b^	15 ± 2

SOR—sorafenib, CIS—cisplatin, 5-FU—5-fluorouracil; ^a^ IC_50_—the concentration that causes 50% growth inhibition; ^b^ colon carcinoma HCT 116; ^c^ lung adenocarcinoma A549.

**Table 3:** DPPH-reducing ability (RA), in vitro inhibition of soybean lipoxygenase (LOX) and lipid peroxidation (LP).

Compd.	RA (%) ^a^		RA (%) ^b^		LOX Inhibition ^a^ (%) (IC_50_ μΜ)	LP Inhibition ^b^ (%)
	20 min	60 min	20 min	60 min		
**3a**	53	33	16	20	19	44
**3b**	5	37	2	2	14	79
**3c**	40	43	22	27	45	70
**3d**	34	31	na	na	43	73
**3e**	16	23	4	9	26	47
**3f**	11	33	na	na	34	87
**3g**	27	46	na	na	17	46
**3h**	na	na	na	na	10	66
**3i**	na	8	na	13	19	32
**3j**	38	40	na	na	(50)	77
**3k**	na	50	na	na	43	65
**7a**	39	100	49	100	36	83
**7b**	84	99	42	100	(100)	62
**7c**	39	100	54	100	(50)	89
**7d**	100	100	55	100	(10)	88
**7e**	46	100	60	100	(43)	86
**7f**	74	100	47	100	(55)	88
**7g**	100	100	66	100	(70)	89
**7h**	52	100	61	100	(41)	67
**7i**	96	73	71	100	(42.5)	84
**7j**	100	95	42	79	(35)	50
**7k**	98	97	72	100	(67.5)	55
NDGA	89	94	83	87	(5.5)	nt
Trolox	nt	nt	nt	nt	nt	88

Concentrations of the tested compounds: ^a^ 1 × 10^−4^ M, ^b^ 5 × 10^−5^ M; na—no activity; nt—not tested.

## Supplementary Materials

Supplementary materials can be accessed at: http://www.mdpi.com/1420-3049/21/12/1629/s1.

## Abbreviations

AAPH	2,2’-azobis(2-amidinopropane) dihydrochloride
BtcCl	1-benzotriazole carboxylic acid chloride
BtH	1*H*-benzo[*d*][1,2,3]triazole
CA	cinnamic acid
CAD	cinnamic acid derivative
CEM	acute lymphoblastic leukemia cell line
CIS	cisplatin
DMEM	Dulbecco′s modified Eagle′s medium
DPPH	1,1-diphenyl-2-picrylhydrazyl
EC_50_	concentration required to reduce virus-induced cytopathogenicity by 50%
FBS	fetal bovine serum
5-FU	5-fluorouracil
H460	lung carcinoma cell line
HEL	human erythroleukemia cell line
HeLa	cervical carcinoma cell line
IC_50_	concentration that causes 50% growth inhibition
L1210	murine lymphocytic leukemia cell line
LOX	soybean lipoxygenase
LP	lipid peroxidation
MCC	minimum cytotoxic concentration, concentration that causes a microscopically detectable alteration of normal cell morphology
MCF-7	breast adenocarcinoma cell line
MR	molar refractivity
MTT	(3-(4,5-dimethylthiazol-2-yl)-2,5-diphenyltetrazolium bromide
NDGA	nordihydroguaiaretic acid
PSA	molecular polar surface area
PG	percentage of growth
PQ	primaquine
RA	DPPH reducing ability
SI	selectivity ratio, MCC to EC_50_ ratio
SOR	sorafenib
SW 620	colon cancer cell line
TEA	triethylamine
Trolox	6-hydroxy-2,5,7,8-tetramethylchroman-2-carboxylic acid
UDA	*Urtica dioica* agglutinin
