# Supplementary material for: Design, Synthesis and Biological Evaluation of Novel Primaquine-Cinnamic Acid Conjugates of the Amide and Acylsemicarbazide Type
Source: Molecules. 2016 Nov 28;21(12):1629. doi: 10.3390/molecules21121629 (PMC6273687; doi:10.3390/molecules21121629)
Supplement: Supplementary file 1 [file molecules-21-01629-s001.pdf]

Supplementary Materials: Design, Synthesis and Biological Evaluation of Novel Primaquine-Cinnamic Acid Conjugates of Amide and Acylsemicarbazide Type

Kristina Pavić, Ivana Perković, Petra Gilja, Filip Kozlina, Katja Ester, Marijeta Kralj, Dominique Schols, Dimitra Hadjipavlou-Litina, Eleni Pontiki and Branka Zorc

Table S1. Analytical and spectral data of PQ-CAD amides **3a**–**k**.

Compd.	Yield (%)		M.p. (°C)	IR (KBr): ν_{max} (cm^{-1})	MS (*m*/*z*)	Molecular Formula (M_r)
	Method A	Method B				
**3a**	65	–	72–74	3387, 3282, 3061, 2927, 2852, 1660, 1614, 1576, 1556, 1516, 1454, 1424, 1387, 1338, 1220, 1203, 1160, 1052, 1031, 977, 902, 863, 822, 791, 766, 680, 626	390.3 [M + 1]$^+$	$C_{24}H_{27}N_3O_2$ (389.49)
**3b**	78	–	oil	(film) 3378, 3054, 2936, 2867, 1735, 1651, 1616, 1578, 1519, 1456, 1424, 1388, 1271, 1220, 1202, 1158, 1052, 1031, 926, 900, 822, 792, 763, 738, 703, 624	404.3 [M + 1]$^+$	$C_{25}H_{29}N_3O_2$ (403.52)
**3c**	66	–	78–80	3255, 3070, 2962, 2930, 2856, 1738, 1652, 1610, 1571, 1517, 1455, 1424, 1385, 1304, 1229, 1168, 1032, 981, 823, 791, 678, 630	420.3 [M + 1]$^+$	$C_{25}H_{29}N_3O_3$ (419.52)
**3d**	70	75	85–88	3355, 3256, 3072, 2964, 2932, 2360, 1738, 1653, 1613, 1555, 1518, 1458, 1425, 1388, 1339, 1298, 1259, 1201, 1166, 1138, 1022, 980, 851, 819, 793, 765, 681, 627, 597	450.5 [M + 1]$^+$	$C_{26}H_{31}N_3O_4$ (449.54)
**3e**	52	69	120–123.5	3367, 3285, 3084, 2962, 1657, 1617, 1583, 1520, 1456, 1421, 1388, 1328, 1277, 1241, 1221, 1200, 1164, 1125, 1052, 1014, 978, 824, 792, 682, 622, 604	480.3 [M + 1]$^+$	$C_{27}H_{33}N_3O_5$ (479.57)
**3f**	57	–	77–79.5	3350, 3253, 3069, 2961, 2934, 1738, 1655, 1620, 1576, 1557, 1520, 1491, 1454, 1425, 1387, 1356, 1334, 1281, 1247, 1200, 1167, 1124, 1096, 1041, 977, 927, 855, 819, 792, 752, 681, 624, 593	434.2 [M + 1]$^+$	$C_{25}H_{27}N_3O_4$ (433.50)
**3g**	79	33	85–87	3358, 3275, 3078, 2958, 2327, 2857, 1740, 1655, 1615, 1558, 1520, 1492, 1457, 1426, 1388, 1343, 1227, 1202, 1168, 1097, 1052, 1013, 982, 903, 823, 791, 736, 709, 677, 628	424.2 [M + 1]$^+$	$C_{24}H_{26}ClN_3O_2$ (423.94)

Table S1. *Cont.*

**3h**	59	–	65–67.5	3357, 3271, 3078, 2960, 2927, 2857, 1739, 1655, 1616, 1578, 1557, 1520, 1487, 1458, 1426, 1388, 1343, 1283, 1226, 1201, 1167, 1093, 1052, 1032, 985, 903, 875, 822, 792, 758, 714, 678, 625	408.1 [M + 1]$^+$	$C_{24}H_{26}FN_3O_2$ (407.48)
**3i**	71	–	48.5–49.5	3262, 3075, 2964, 2861, 1661, 1618, 1561, 1520, 1454, 1387, 1335, 1221, 1166, 1126, 975, 803, 864, 820, 793, 735, 688, 625, 584, 513	458.3 [M + 1]$^+$	$C_{25}H_{26}F_3N_3O_2$ (457.49)
**3j**	73	–	82–84	3359, 3262, 3075, 2963, 2931, 2856, 1738, 1655, 1615, 1577, 1560, 1519, 1456, 1424, 1386, 1326, 1227, 1203, 1163, 1130, 1110, 1069, 1050, 1015, 979, 957, 903, 880, 829, 792, 719, 681, 623, 596	458.2 [M + 1]$^+$	$C_{25}H_{26}F_3N_3O_2$ (457.49)
**3k**	74	–	148–150	3395, 3286, 3095, 2964, 2934, 2861, 1663, 1623, 1576, 1521, 1456, 1423, 1334, 1341, 1278, 1224, 1174, 1136, 1052, 1031, 978, 940, 899, 868, 845, 822, 792, 683, 624	526.4 [M + 1]$^+$	$C_{26}H_{25}F_6N_3O_2$ (525.49)

Table S2. ^{1}H- and ^{13}C-NMR spectra of PQ-CAD amides **3a**–**k**.

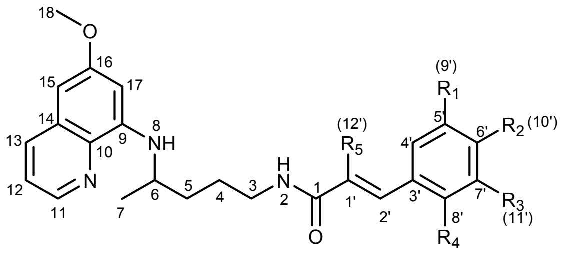

3a-k

Compd.	R$_1$	R$_2$	R$_3$	R$_4$	R$_5$	^{1}H-NMR (DMSO-d_6, δ ppm, J/Hz)	^{13}C-NMR (DMSO-d_6, δ ppm, J/Hz)
**3a**	H	H	H	H	H	8.55–8.53 (dd, 1H, 11, J = 1.43, 4.13), 8.13 (t, 1H, 2, J = 5.69), 8.09–8.06 (dd, 1H, 13, J = 1.43, 8.28), 7.55 (d, 2H, 4', 8', J = 6.49), 7.45–7.34 (m, 5H, 12, 2', 5', 6', 7'), 6.61 (d, 1H, 1', J = 15.82), 6.47 (s, 1H, 17), 6.28 (s, 1H, 15), 6.15 (d, 1H, 8, J = 8.73), 3.81 (s, 3H, 18), 3.66 (m, 1H, 6), 3.21 (m, 2H, 3), 1.64 (m, 4H, 4, 5), 1.22 (d, 3H, 7, J = 6.25)	164.78 (1), 159.01 (16), 144.64 (9), 144.23 (11), 138.40 (2'), 134.96 (10), 134.80 (13), 134.52 (3'), 129.58 (14), 129.33 (6'), 128.89 (5', 7'), 127.44 (4', 8'), 122.32 (12), 122.10 (1'), 96.12 (17), 91.61 (15), 54.96 (18), 47.01 (6), 39.02 (3), 33.54 (5), 26.03 (4), 20.23 (7)

Table S2. *Cont.*

**3b**	H	H	H	H	$^{12'}CH_3$	8.55–8.53 (dd, 1H, 11, *J* = 1.63, 4.19), 8.09–8.03 (m, 2H, 2, 13), 7.45–7.28 (m, 6H, 12, 4'–8'), 7.18 (s, 1H, 2'), 6.48 (s, 1H, 17), 6.28 (s, 1H, 15), 6.16–6.14 (d, 1H, 8, *J* = 8.75), 3.81 (s, 3H, 18), 3.70–3.63 (m, 1H, 6), 3.23–3.17 (m, 2H, 3), 1.99 (s, 3H, 12'), 1.73–1.56 (m, 4H, 4, 5), 1.24–1.23 (d, 3H, 7, *J* = 6.29)	168.71 (1), 159.02 (16), 144.58 (9), 144.27 (11), 136.14 (3'), 134.80 (13), 134.54 (10), 132.68 (1'), 131.87 (2′), 129.58 (14), 129.17 (5', 7'), 128.35 (4', 8'), 127.56 (6'), 122.10 (12), 96.11 (17), 91.58 (15), 54.96 (18), 47.04 (6), 39.02 (3), 33.43 (5), 25.95 (4), 20.19 (7), 14.29 (12')
**3c**	H	$^{10'}OCH_3$	H	H	H	8.54–8.53 (dd, 1H, 11, *J* = 1.5, 4.2), 8.09–8.05 (dd, 1H, 13, *J* = 1.4, 8.3), 8.02–7.98 (t, 1H, 2, *J* = 5.4), 7.50–7.47 (d, 2H, 4', 8', *J* = 8.69), 7.44–7.40 (m, 1H, 12), 7.38–7.32 (d, 1H, 2', *J* = 15.75), 6.98–6.95 (d, 2H, 5', 7', *J* = 8.70), 6.48–6.42 (m, 2H, 17, 1'), 6.28 (s, 1H, 15), 6.15–6.12 (d, 1H, 8, *J* = 8.74), 3.81 (s, 3H, 18), 3.78 (s, 3H, 10'), 3.65 (m, 1H, 6), 3.21–3.19 (m, 2H, 3), 1.7–1.56 (m, 4H, 4, 5), 1.23–1.21 (d, 3H, 7, *J* = 6.27)	165.05 (1), 160.18 (6′), 158.98 (16), 144.61 (9), 144.18 (11), 138.02 (2'), 134.74 (13), 134.49 (10), 129.53 (14), 128.93 (4', 8'), 127.50 (3'), 122.03 (12), 119.85 (1'), 114.30 (5', 7'), 96.08 (17), 91.61 (15), 55.18 (18), 54.93 (10'), 47.00 (6), 39.02 (3), 33.54 (5), 26.03 (4), 20.19 (7)
**3d**	H	$^{10'}OCH_3$	$^{11'}OCH_3$	H	H	8.54–8.53 (dd, 1H, 11, *J* = 1.62, 4.19), 8.09–8.05 (dd, 1H, 13, *J* = 1.56, 8.29), 8.00–7.96 (t, 1H, 2, *J* = 5.52), 7.44–7.40 (m, 1H, 12, *J* = 4.20, 8.25), 7.36–7.31 (d, 1H, 2', *J* = 15.73), 7.14–7.08 (m, 2H, 4', 8'), 6.98–6.96 (d, 1H, 1', *J* = 8.32), 6.51–6.45 (m, 2H, 17, 7'), 6.28 (s, 1H, 15), 6.15–6.12 (d, 1H, 8, *J* = 8.76), 3.81–3.78 (m, 9H, 18, 10', 11'), 3.65 (m, 1H, 6), 3.23–3.19 (m, 2H, 3), 1.70–1.56 (m, 4H, 4, 5), 1.23–1.21 (d, 2H, 7, *J* = 6.29)	165.05 (1), 158.97 (16), 149.98 (5'), 148.85 (6'), 144.61 (9), 144.18 (11), 138.36 (2'), 134.73 (13), 134.49 (10), 129.53 (14), 127.75 (3'), 122.03 (12), 121.20 (8'), 120.05 (1'), 111.74 (7'), 109.96 (4'), 96.08 (17), 91.61 (15), 55.49 (11'), 55.37 (10'), 54.92 (18), 47.00 (6), 39.02 (3), 33.55 (5), 26.02 (4), 20.20 (7)
**3e**	$^{9'}OCH_3$	$^{10'}OCH_3$	$^{11'}OCH_3$	H	H	8.54–8.53 (d, 1H, 11, *J* = 2.85), 8.09–8.03 (m, 2H, 2, 13), 7.44–7.40 (m, 1H, 12), 7.37–7.32 (d, 1H, 2', *J* = 15.68), 6.88 (s, 2H, 4', 8'), 6.54–6.52 (d, 1H, 1', *J* = 15.69), 6.47 (s, 1H, 17), 6.28 (s, 1H, 15), 6.15–6.12 (d, 1H, 8, *J* = 8.69), 3.81 (s, 9H, 18, 9', 11'), 3.68 (s, 4H, 6, 10'), 3.21–3.19 (m, 2H, 3), 1.69–1.65 (m, 4H, 4, 5), 1.23–1.22 (d, 3H, 7, *J* = 6.15)	164.83 (1), 158.97 (16), 153.00 (5', 7'), 144.62 (9), 144.18 (11), 138.57 (6'), 138.46 (2'), 134.74 (13), 134.49 (10), 130.55 (14), 129.53 (3'), 122.04 (12), 121.66 (1'), 104.87 (4', 8'), 96.08 (17), 91.61 (15), 60.02 (10'), 55.82 (9', 11'), 54.92 (18), 46.99 (6), 39.02 (3), 33.56 (5), 25.98 (4), 20.20 (7)

Table S2. *Cont.*

**3f**	H	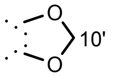		H	H	8.53–8.51 (dd, 1H, 11, *J* = 1.61, 4.18), 8.07–8.04 (dd, 1H, 13, *J* = 1.57, 8.29), 7.99–7.95 (t, 1H, 2, *J* = 5.52), 7.43–7.39 (m, 1H, 12), 7.33–7.28 (d, 1H, 2'), 7.11 (s, 1H, 4'), 7.05–7.02 (dd, 1H, 8', *J* = 1.46, 8.11), 6.93–6.91 (d, 1H, 1', *J* = 8.00), 6.46–6.40 (m, 2H, 17, 7'), 6.27 (s, 1H, 15), 6.14–6.11 (d, 1H, 8, *J* = 8.77), 6.04 (s, 2H, 10'), 3.80 (s, 3H, 18), 3.64 (m, 1H, 6), 3.21–3.15 (m, 2H, 3), 1.69–1.51 (m, 4H, 4, 5), 1.22–1.20 (d, 2H, 7, *J* = 6.29)	164.93 (1), 158.97 (16), 148.29 (5'), 147.85 (6'), 144.61 (9), 144.18 (11), 138.13 (2'), 134.73 (13), 134.49 (10), 129.53 (14), 129.33 (3'), 123.00 (8'), 122.05 (12), 120.42 (1'), 108.49 (7'), 106.11 (4'), 101.33 (10'), 96.08 (17), 91.61 (15), 54.93 (18), 46.99 (6), 39.02 (3), 33.53 (5), 26.01 (4), 20.19 (7)
**3g**	H	Cl	H	H	H	8.54–8.53 (dd, 1H, 11, *J* = 1.60, 4.19), 8.13–8.05 (m, 2H, 2, 13), 7.58–7.56 (d, 2H, 4', 8', *J* = 8.54), 7.47–7.37 (m, 4H, 5, 12, 2', 7'), 7.63–7.58 (d, 1H, 1', *J* = 15.82), 7.47 (s, 1H, 17), 6.28 (s, 1H, 15), 6.15–6.12 (d, 1H, 8, *J* = 8.79), 3.81 (s, 3H, 18), 3.65 (m, 1H, 6), 3.22–3.20 (m, 2H, 3), 1.72–1.53 (m, 4H, 4, 5), 1.23–1.21 (d, 3H, 7, *J* = 6.28)	164.56 (1), 158.97 (16), 144.60 (9), 144.18 (11), 136.97 (2'), 134.73 (13), 134.49 (10), 133.91 (6'), 133.68 (3'), 129.53 (14), 129.08 (5', 7'), 128.85 (4', 8'), 123.14 (12), 122.03 (1'), 96.08 (17), 91.61 (15), 54.92 (18), 46.98 (6), 39.02 (3), 33.51 (5), 26.95 (4), 20.19 (7)
**3h**	H	H	H	F	H	8.55–8.53 (dd, 1H, 11, *J* = 1.60, 4.19), 8.23–8.19 (t, 1H, 2, *J* = 5.50), 8.09–8.05 (dd, 1H, 13, *J* = 1.55, 8.28), 7.66–7.61 (t, 1H, 6', *J* = 6.49), 7.50–7.39 (m, 3H, 12, 2', 8'), 7.30–7.23 (m, 2H, 5', 7'), 6.73–6.68 (d, 1H, 1', *J* = 15.94), 6.47 (s, 1H, 17), 6.28 (s, 1H, 15), 6.15–6.13 (d, 1H, 8, *J* = 8.79), 3.81 (s, 3H, 18), 3.65 (m, 1H, 6), 3.25–3.21 (m, 2H, 3), 1.73–1.53 (m, 4H, 4, 5), 1.24–1.21 (d, 3H, 7, *J* = 6.28)	164.48 (1), 162.02–158.71 (d, 8', *J* = 250.43), 158.97 (16), 144.61 (9), 144.18 (11), 134.73 (13), 134.49 (10), 131.14–131.02 (d, 6', *J* = 8.58), 130.72 (2'), 129.53 (14), 129.03–128.99 (d, 5', *J* = 3.26), 125.17–125.09 (d, 4', *J* = 6.18), 124.97 (1'), 122.61–122.41 (d, 3', *J* = 12.66), 122.03 (12), 116.12–115.83 (d, 7', *J* = 23.47), 96.08 (17), 91.61 (15), 54.91 (18), 46.98 (6), 39.02 (3), 33.51 (5), 25.91 (4), 20.19 (7)
**3i**	H	H	$^{11'}CF_3$	H	H	8.55–8.53 (dd, 1H, 11, *J* = 1.46, 4.10), 8.15–8.11 (t, 1H, 2, *J* = 5.51), 8.08–8.05 (dd, 1H, 13, *J* = 1.56, 8.27), 7.89–7.84 (m, 2H, 6', 8'), 7.73–7.62 (m, 2H, 4', 5'), 7.52–7.47 (d, 1H, 2', *J* = 15.84), 7.44–7.40 (m, 1H, 12), 6.77–6.72 (d, 1H, 1', *J* = 15.85), 6.47 (s, 1H, 17), 6.29 (s, 1H, 15), 6.16–6.13 (d, 1H, 8, *J* = 8.76), 3.81 (s, 3H, 18), 3.66 (m, 1H, 6), 3.23–3.21 (m, 2H, 3), 1.72–1.57 (m, 4H, 4, 5), 1.24–1.22 (d, 3H, 7, *J* = 6.25)	164.34 (1), 158.97 (16), 144.61 (9), 144.18 (11), 136.62 (2'), 136.17 (3'), 134.73 (13), 134.50 (10), 132.28–122.55 (q, 11', *J* = 244.60), 131.20 (4'), 130.28–128.83 (q, 7', *J* = 31.73), 129.96 (5'), 129.44 (14), 125.60–125.46 (q, 6', *J* = 3.70), 124.48 (1'), 123.75–123.60 (q, 8', *J* = 3.71), 122.03 (12), 96.09 (17), 91.60 (15), 54.91 (18), 46.98 (6), 39.02 (3), 33.50 (5), 25.91 (4), 20.20 (7)

Table S2. *Cont.*

**3j**	H	[10']CF_3	H	H	H	8.55–8.53 (dd, 1H, 11, *J* = 1.61, 4.19), 8.24–8.21 (t, 1H, 2, *J* = 5.56), 8.09–8.06 (dd, 1H, 13, *J* = 1.57, 8.30), 7.76 (s, 4H, 4', 5', 7', 8'), 7.50–7.45 (d, 1H, 2', *J* = 15.87), 7.45–7.40 (m, 1H, 12), 6.76–6.71 (d, 1H, 1', *J* = 15.84), 6.47 (s, 1H, 17), 6.29 (s, 1H, 15), 6.16–6.13 (d, 1H, 8, *J* = 8.80), 3.81 (s, 3H, 18), 3.66 (m, 1H, 6), 3.25–3.21 (m, 2H, 3), 1.73–1.53 (m, 4H, 4, 5), 1.24–1.21 (d, 3H, 7, *J* = 6.28)	164.31 (1), 159.00 (16), 144.63 (9), 144.23 (11), 139.07 (3'), 136.74 (2'), 134.79 (13), 134.52 (10), 129.57 (14), 129.71–128.44 (q, 6', *J* = 31.56), 129.51–118.69 (q, 10', *J* = 272.14), 128.07 (4', 8'), 125.82–125.67 (q, 5', 7', *J* = 3.80), 125.12 (1'), 122.09 (12), 96.12 (17), 91.60 (15), 54.95 (18), 46.99 (6), 39.02 (3), 33.52 (5), 23.96 (4), 20.23 (7)
**3k**	[9']CF_3	H	[11']CF_3	H	H	8.54 (s, 1H, 11), 8.26 (s, 2H, 4', 8'), 8.18 (t, 1H, 2, *J* = 5.21), 8.08–8.07 (m, 2H, 13, 6'), 7.60–7.58 (d, 1H, 2', *J* = 15.82), 7.44–7.42 (m, 1H, 12), 6.91–6.88 (d, 1H, 1', *J* = 15.90), 6.47 (s, 1H, 17), 6.28 (s, 1H, 15), 6.16–6.14 (d, 1H, 8, *J* = 8.54), 3.80 (s, 3H, 18), 3.66 (m, 1H, 6), 3.23 (m, 2H, 3), 1.70–1.58 (m, 4H, 4, 5), 1.23–1.22 (d, 3H, 7, *J* = 5.99)	164.07 (1), 159.00 (16), 144.64 (9), 144.24 (11), 137.97 (3'), 135.16 (2'), 134.81 (13), 134.53 (10), 131.14–130.48 (q, 5', 7', *J* = 33.83), 129.58 (14), 127.84 (4', 8'), 126.61 (6'), 125.94–120.54 (q, 9', 11', *J* = 272.30), 122.23 (1'), 122.11 (12), 96.14 (17), 91.59 (15), 54.95 (18), 46.99 (6), 39.02 (3), 33.51 (5), 25.92 (4), 20.25 (7)

Table S3. Analytical and spectral data of PQ-CAD semicarbazides **7a–k**.

Compd.	Yield (%)	M.p. (°C)	IR (KBr): ν_{max} (cm^{-1})	MS (*m*/*z*)	Molecular formula (M_r)
**7a**	56	188–190 (decomp.)	3302, 3236, 3038, 2936, 1696, 1628, 1578, 1596, 1458, 1388, 1424, 1356, 1222, 1156, 1054, 978, 862, 822, 790, 762, 726, 678, 658, 632, 556, 488	448.3 [M + 1]$^+$	$C_{25}H_{29}N_5O_3$ (447.53)
**7b**	53	65.5 (decomp.)	3265, 2961, 2935, 1652, 1617, 1576, 1520, 1454, 1423, 1387, 1336, 1258, 1239, 1220, 1203, 1158, 1051, 1031, 1004, 928, 910, 822, 791, 762, 709, 695, 625, 589, 515	462.3 [M + 1]$^+$	$C_{26}H_{31}N_5O_3$ (461.56)
**7c**	60	89.5 (decomp.)	3248, 2934, 1654, 1604, 1575, 1517, 1456, 1424, 1386, 1251, 1167, 1029, 980, 824, 791, 628, 521	478.3 [M + 1]$^+$	$C_{26}H_{31}N_5O_4$ (477.56)
**7d**	57	200.5–201.5 (decomp.)	3375, 3213, 3084, 3003, 2937, 1668, 1628, 1596, 1559, 1515, 1456, 1421, 1387, 1356, 1293, 1262, 1235, 1202, 1171, 1138, 1052, 1022, 977, 939, 856, 816, 788, 767, 710, 679, 622, 594, 560, 460	508.3 [M + 1]$^+$	$C_{27}H_{33}N_5O_5$ (507.58)
**7e**	62	90.5 (decomp.)	3250, 2937, 1654, 1618, 1582, 1508, 1454, 1420, 1388, 1326, 1266, 1240, 1221, 1203, 1155, 1126, 1051, 1031, 1003, 976, 822, 791, 677, 624, 585, 526, 512, 464	538.3 [M + 1]$^+$	$C_{28}H_{35}N_5O_6$ (537.61)
**7f**	49	75.5 (decomp.)	3250, 2335, 2361, 1654, 1618, 1577, 1560, 1521, 1490, 1448, 1388, 1252, 1202, 1158, 1100, 1037, 976, 929, 819, 791, 670, 625, 592, 518	492.3 [M + 1]$^+$	$C_{26}H_{29}N_5O_5$ (491.54)

Table S3. *Cont.*

**7g**	59	185–187.5	3337, 3230, 3037, 2966, 2937, 2362, 2343, 1697, 1661, 1625, 1592, 1521, 1490, 1458, 1425, 1408, 1391, 1354, 1290, 1266, 1239, 1221, 1199, 1161, 1090, 1052, 1010, 982, 945, 899, 866, 819, 790, 726, 676, 628, 495	482.2 [M + 1]$^+$	$C_{25}H_{28}ClN_5O_3$ (481.97)
**7h**	89	91–93 (decomp.)	3250, 2964, 1654, 1618, 1578, 1520, 1457, 1388, 1221, 1159, 1052, 982, 822, 791, 757	466.1 [M + 1]$^+$	$C_{25}H_{28}FN_5O_3$ (465.52)
**7i**	74	86–88 (decomp.)	3254, 2964, 1659, 1618, 1577, 1521, 1456, 1424, 1388, 1334, 1222, 1199, 1166, 1126, 1075, 976, 900, 822, 792, 694, 660, 625, 561, 514	516.2 [M + 1]$^+$	$C_{26}H_{28}F_3N_5O_3$ (515.53)
**7j**	67	123 (decomp.)	3266, 1695, 1664, 1612, 1525, 1469, 1425, 1390, 1327, 1235, 1169, 1118, 1067, 834, 789, 728, 631, 592	516.2 [M + 1]$^+$	$C_{26}H_{28}F_3N_5O_3$ (515.53)
**7k**	56	118–119.5 (decomp.)	3393, 3335, 3220, 3020, 2942, 2363, 1647, 1617, 1579, 1521, 1459, 1425, 1384, 1340, 1280, 1224, 1175, 1135, 1053, 971, 942, 898, 847, 823, 792, 730, 683, 629, 599, 561, 519, 466	584.3 [M + 1]$^+$	$C_{27}H_{27}F_6N_5O_3$ (583.53)

Table S4. ^{1}H and ^{13}C NMR spectra of PQ-CAD semicarbazides **7a–k**.

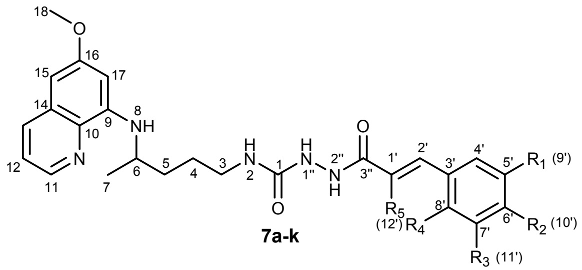

Compd.	R$_1$	R$_2$	R$_3$	R$_4$	R$_5$	^{1}H-NMR (DMSO-d_6, δ ppm, *J*/Hz)	^{13}C-NMR (DMSO-d_6, δ ppm, *J*/Hz)
**7a**	H	H	H	H	H	9.75 (s, 1H, 2''), 8.54–8.53 (dd, 1H, 11, *J* = 1.41, 4.11), 8.09–8.06 (dd, 1H, 13, *J* = 1.30, 8.25), 7.85 (s, 1H, 1''), 7.58–7.36 (m, 7H, 12, 2', 4'–8'), 6.65–6.60 (d, 1H, 1', *J* = 15.90), 6.48–6.44 (m, 2H, 2, 17), 6.28 (s, 1H, 15), 6.13–6.10 (d, 1H, 8, *J* = 8.62), 3.82 (s, 3H, 18), 3.64 (m, 1H, 6), 3.06–3.04 (m, 2H, 3), 1.63–1.47 (m, 4H, 4, 5), 1.22–1.20 (d, 3H, 7, *J* = 6.24)	164.65 (3''), 158.98 (16), 157.92 (1), 144.61 (9), 144.20 (11), 139.46 (2'), 134.74 (13), 134.67 (10), 134.49 (3'), 129.60 (6'), 129.53 (14), 128.92 (5', 7'), 127.50 (4', 8'), 122.04 (12), 119.94 (1'), 96.07 (17), 91.61 (15), 54.95 (18), 47.01 (6), 39.02 (3), 33.41 (5), 26.65 (4), 20.18 (7)

Table S4. *Cont.*

**7b**	H	H	H	H	12'CH3	9.67 (s, 1H, 2''), 8.54–8.53 (dd, 1H, 11, *J* = 1.57, 4.15), 8.08–8.07 (dd, 1H, 13, *J* = 1.49, 8.28), 7.67 (s, 1H, 1''), 7.43–7.30 (m, 7H, 12, 2', 4'–8'), 6.48–6.45 (m, 2H, 2, 17), 6.28 (s, 1H, 15), 6.13–6.11 (d, 1H, 8, *J* = 8.75), 4.05–3.82 (s, 3H, 18), 3.65–3.63 (m, 1H, 6), 3.08–3.02 (m, 2H, 3), 2.02 (s, 3H, 12'), 1.66–1.47 (m, 4H, 4, 5), 1.22–1.21 (d, 3H, 7, *J* = 6.29)	168.89 (3''), 159.00 (16), 158.36 (1), 144.63 (9), 144.24 (11), 135.83 (3'), 134.78 (13), 134.51 (10), 133.23 (2'), 130.74 (1'), 129.56 (14), 129.22 (5', 7'), 128.42 (4', 8'), 127.80 (6'), 122.08 (12), 96.10 (17), 91.61 (15), 54.97 (18), 47.02 (6), 39.02 (3), 33.43 (5), 26.73 (4), 20.21 (7), 14.15 (12')
**7c**	H	10'OCH3	H	H	H	9.64 (s, 1H, 2''), 8.54–8.52 (dd, 1H, 11, *J* = 1.45, 4.10), 8.08–8.06 (d, 1H, 13, *J* = 7.07), 7.80 (s, 1H, 1''), 7.53–7.42 (m, 4H, 12, 2', 4', 8'), 6.99–6.97 (d, 2H, 5', 7', *J* = 8.66), 6.49–6.45 (m, 3H, 2, 17, 1'), 6.27 (s, 1H, 15), 6.10 (s, 1H, 8), 3.82 (s, 3H, 18), 3.78 (s, 3H, 10'), 3.62 (m, 1H, 6), 3.04 (m, 2H, 3), 1.63–1.46 (m, 4H, 4, 5), 1.21–1.19 (d, 3H, 7, *J* = 6.23)	165.24 (3''), 160.56 (6'), 159.06 (16), 158.17 (1), 144.69 (9), 144.35 (11), 139.41 (2'), 134.90 (13), 134.56 (10), 129.65 (14), 129.28 (4', 8'), 127.30 (3'), 122.19 (12), 117.37 (1'), 114.49 (5', 7'), 96.19 (17), 91.71 (15), 55.34 (18), 55.07 (10'), 47.11 (6), 39.02 (3), 33.49 5), 26.77 (4), 20.29 (7)
**7d**	H	10'OCH3	11'OCH3	H	H	9.62–9.61 (d, 1H, 2'', *J* = 1.73), 8.54–8.52 (dd, 1H, 11, *J* = 1.39, 4.06), 8.08–8.06 (dd, 1H, 13, *J* = 1.29, 8.29), 7.81 (s, 1H, 1''), 7.44–7.40 (m, 2H, 12, 2'), 7.15–7.12 (m, 2H, 4', 8'), 7.00–6.98 (d, 1H, 1', *J* = 8.28), 6.53–6.46 (m, 3H, 2, 17, 7'), 6.26 (s, 1H, 15), 6.11–6.09 (d, 1H, 8, *J* = 8.73), 3.81–3.78 (m, 9H, 18, 10', 11'), 3.62 (m, 1H, 6), 3.04–3.02 (m, 2H, 3), 1.54–1.51 (m, 4H, 4, 5), 1.21–1.19 (d, 3H, 7, *J* = 6.25)	165.27 (3''), 159.06 (16), 158.19 (1), 150.32 (5'), 148.93 (6'), 144.69 (9), 144.36 (11), 139.74 (2'), 134.91 (13), 134.57 (10), 129.66 (14), 127.55 (3'), 122.21 (12), 121.48 (8'), 117.64 (1'), 111.81 (7'), 110.20 (4'), 96.19 (17), 91.69 (15), 55.62 (10'), 55.52 (18), 55.08 (11'), 47.11 (6), 39.02 (3), 33.50 (5), 26.80 (4), 20.30 (7)
**7e**	9'OCH3	10'OCH3	11'OCH3	H	H	9.64–9.63 (d, 1H, 2'', *J* = 1.91), 8.53–8.52 (dd, 1H, 11, *J* = 1.51, 4.16), 8.07–8.05 (dd, 1H, 13, *J* = 1.41, 8.28), 7.83 (s, 1H, 1''), 7.45–7.40 (m, 2H, 12, 2'), 6.90 (s, 2H, 4', 8'), 6.59–6.55 (d, 1H, 1', *J* = 15.80), 6.46–6.43 (m, 2H, 2, 17), 6.26 (s, 1H, 15), 6.11–6.09 (d, 1H, 8, *J* = 8.78), 3.81 (s, 3H, 18), 3.80 (s, 6H, 9', 11'), 3.68 (s, 3H, 10'), 3.63 (m, 1H, 6), 3.04–3.02 (m, 2H, 3), 1.61–1.51 (m, 4H, 4, 5), 1.21–1.19 (d, 3H, 7, *J* = 6.25)	164.92 (3''), 159.05 (16), 158.05 (1), 153.14 (5', 7'), 144.69 (9), 144.33 (11), 139.82 (2'), 138.89 (6'), 134.88 (13), 134.56 (10), 130.38 (14), 129.64 (3'), 122.17 (12), 119.26 (1'), 105.09 (4', 8'), 96.17 (17), 91.69 (15), 60.16 (10'), 55.96 (9', 11'), 55.05 (18), 47.10 (6), 39.02 (3), 33.49 (5), 26.76 (4), 20.28 (7)
**7f**	H	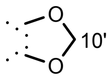	H	H		9.64 (s, 1H, 2''), 8.53 (d, 1H, 11, *J* = 2.84), 8.08–8.06 (d, 1H, 13, *J* = 8.05), 7.82 (s, 1H, 1''), 7.43–7.39 (m, 2H, 12, 2'), 7.15 (s, 1H, 4'), 7.09–7.07 (d, 1H, 8', *J* = 8.05), 6.96–6.94 (d, 1H, 1', *J* = 7.96), 6.48–6.44 (m, 3H, 2, 17, 7'), 6.26 (s, 1H, 15), 6.11–6.09 (d, 1H, 8, *J* = 7.59), 6.06 (s, 3H, 10'), 3.82 (s, 3H, 18), 3.62 (m, 1H, 6), 3.04 (m, 2H, 3), 1.60–1.46 (m, 4H, 4, 5), 1.12–1.19 (d, 3H, 7, *J* = 6.13)	165.45 (3''), 159.43 (16), 158.49 (1), 149.08 (5'), 148.38 (6'), 145.05 (9), 144.69 (11), 139.84 (2'), 135.25 (13), 134.93 (10), 130.01 (14), 129.50 (3'), 123.83 (8'), 122.54 (12), 118.33 (1'), 109.05 (7'), 106.66 (4'), 101.92 (10'), 96.55 (17), 92.06 (15), 55.42 (18), 47.47 (6), 39.02 (3), 33.86 (5), 27.14 (4), 20.65 (7)

Table S4. *Cont.*

**7g**	H	Cl	H	H	H	9.79 (s, 1H, 2''), 8.53–8.52 (dd, 1H, 11, *J* = 1.06, 4.11), 8.08–8.05 (dd, 1H, 13, *J* = 1.30, 8.25), 7.88 (s, 1H, 1''), 7.61–7.59 (d, 2H, 4', 8', *J* = 8.47), 7.50–7.45 (m, 3H, 2', 5', 7'), 7.43–7.40 (m, 1H, 12), 6.64–6.60 (d, 1H, 1', *J* = 15.89), 6.51–6.48 (t, 1H, 2, *J* = 5.44), 6.46 (s, 1H, 17), 6.25 (s, 1H, 15), 6.11–6.09 (d, 1H, 8, *J* = 8.71), 3.81 (s, 3H, 18), 3.62 (m, 1H, 6), 3.04 (m, 2H, 3), 1.61–1.51 (m, 4H, 4, 5), 1.20–1.19 (d, 3H, 7, *J* = 6.25)	164.63 (3''), 159.05 (16), 158.03 (1), 144.68 (9), 144.33 (11), 138.28 (2'), 134.89 (13), 134.56 (10), 134.18 (6), 133.68 (3'), 129.64 (14), 129.35 (4', 8'), 129.09 (5', 7'), 122.19 (12), 120.75 (1'), 96.17 (17), 91.66 (15), 55.06 (18), 47.08 (6), 39.02 (3), 33.47 (5), 26.77 (4), 20.28 (7)
**7h**	H	H	H	F	H	9.85 (s, 1H, 2''), 8.54–8.53 (dd, 1H, 11, *J* = 1.50, 4.10), 8.08–8.06 (dd, 1H, 13, *J* = 1.42, 8.22), 7.87 (s, 1H, 1''), 7.67–7.65 (t, 1H, 6', *J* = 7.22), 7.57–7.55 (d, 1H, 2', *J* = 16.02), 7.45–7.41 (m, 2H, 12, 4'), 7.30–7.26 (m, 2H, 5', 7'), 6.76–6.73 (d, 1H, 1', *J* = 16.03), 6.48 (s, 2H, 2, 17), 6.28 (s, 1H, 15), 6.12–6.11 (d, 1H, 8, *J* = 8.70), 3.83 (s, 3H, 18), 3.64 (m, 1H, 6), 3.07 (m, 2H, 3), 1.65–1.49 (m, 4H, 4, 5), 1.22–1.21 (d, 3H, 7, *J* = 6.28)	164.43 (3''), 161.30–159.64 (d, 8', *J* = 250.54), 158.99 (16), 157.86 (1), 144.62 (9), 144.21 (11), 134.74 (13), 134.50 (10), 131.90 (2'), 131.47–131.41 (d, 6', *J* = 8.67), 129.54 (14), 129.22–129.20 (d, 5', *J* = 2.69), 124.98 (1'), 122.90–122.86 (d, 4', *J* = 6.35), 122.35–122.28 (d, 3', *J* = 11.54), 122.04 (12), 116.13–115.99 (d, 7', *J* = 21.68), 96.09 (17), 91.04 (15), 54.95 (18), 47.03 (6), 39.02 (3), 33.43 (5), 26.64 (4), 20.19 (7)
**7i**	H	H	$^{11'}CF_3$	H	H	9.77 (s, 1H, 2''), 8.54 (d, 1H, 11, *J* = 2.58), 8.08–8.06 (d, 1H, 13, *J* = 7.91), 7.92–7.88 (m, 3H, 6', 8', 1''), 7.75–7.73 (d, 1H, 4', *J* = 7.53), 7.68–7.65 (t, 1H, 5', *J* = 7.55), 7.61–7.59 (d, 1H, 2', *J* = 15.88), 7.43–7.41 (m, 1H, 12), 6.78–6.76 (d, 1H, 1', *J* = 15.89), 6.47 (s, 2H, 2, 17), 6.28 (s, 1H, 15), 6.12–6.11 (d, 1H, 8, *J* = 8.50), 3.83 (s, 3H, 18), 3.64 (m, 1H, 6), 3.07–3.06 (m, 2H, 3), 1.65–1.49 (m, 4H, 4, 5), 1.22–1.21 (d, 3H, 7, *J* = 6.00)	164.18 (3''), 158.99 (16), 157.81 (1), 144.62 (9), 144.20 (11), 137.75 (2'), 135.90 (3'), 134.74 (13), 134.50 (10), 132.14–127.87 (q, 11', *J* = 214.50), 131.15 (4'), 130.07–129.41 (q, 7', *J* = 30.02), 130.06 (5'), 129.53 (14), 125.84 (6'), 123.98 (8'), 122.11 (12), 122.03 (1'), 96.08 (17), 91.64 (15), 54.94 (18), 47.03 (6), 39.02 (3), 33.43 (5), 26.64 (4), 20.18 (7)
**7j**	H	$^{10'}CF_3$	H	H	H	9.85 (s, 1H, 2''), 8.54–8.53 (d, 1H, 11, *J* = 3.71), 8.08–8.06 (d, 1H, 13, *J* = 8.07), 7.90 (s, 1H, 1''), 7.78 (s, 4H, 4', 5', 7', 8'), 7.59–7.56 (d, 1H, 2', *J* = 15.92), 7.43–7.41 (m, 1H, 12), 6.77–6.74 (d, 1H, 1', *J* = 15.90), 6.47 (s, 2H, 2, 17), 6.28 (s, 1H, 15), 6.12–6.11 (d, 1H, 8, *J* = 8.58), 3.83 (s, 3H, 18), 3.64 (m, 1H, 6), 3.07–3.05 (m, 2H, 3), 1.65–1.47 (m, 4H, 4, 5), 1.22–1.21 (d, 3H, 7, *J* = 6.06)	164.10 (3''), 158.98 (16), 157.80 (1), 144.61 (9), 144.19 (11), 138.73 (3'), 137.75 (2'), 134.73 (10), 134.49 (13), 129.64–129.01 (q, 6', *J* = 31.19), 129.53 (14), 128.13 (4', 8'), 126.81–121.32 (q, 10', *J* = 271.67), 125.76 (5', 7'), 122.77 (1'), 122.02 (12), 96.07 (17), 91.63 (15), 54.93 (18), 47.01 (6), 39.02 (3), 33.42 (5), 26.63 (4), 20.17 (7)
**7k**	$^{9'}CF_3$	H	$^{11'}CF_3$	H	H	9.81 (s, 1H, 2''), 8.55–8.53 (dd, 1H, 11, *J* = 1.62, 4.19), 8.29 (s, 2H, 4', 8'), 8.12 (s, 1H, 6'), 8.09–8.07 (dd, 1H, 13, *J* = 1.58, 8.31), 7.99 (s, 1H, 1''), 7.72–7.68 (d, 1H, 2', *J* = 15.92), 7.77–7.41 (m, 1H, 12), 6.94–6.90 (d, 1H, 1', *J* = 15.97), 6.51–6.47 (m, 2H, 2, 17), 6.28 (s, 1H, 15), 6.14–6.11 (d, 1H, 8, *J* = 8.74), 3.82 (s, 3H, 18), 3.64 (m, 1H, 6), 3.06 (m, 2H, 3), 1.66–1.48 (m, 4H, 4, 5), 1.22–1.21 (d, 3H, 7, *J* = 6.28)	163.86 (3''), 159.02 (16), 157.78 (1), 144.65 (9), 144.27 (11), 137.67 (3'), 136.28 (2'), 134.83 (13), 134.53 (10), 131.40–130.40 (q, 5', 7', *J* = 33.53), 129.60 (14), 128.22 (4', 8'), 127.28–119.14 (q, 9', 11', *J* = 272.61), 125.53 (6'), 124.23 (1'), 122.13 (12), 96.13 (17), 91.61 (15), 55.01 (18), 47.04 (6), 39.02 (3), 33.45 (5), 26.74 (4), 20.25 (7)
